# EasyDAM_V3: Automatic Fruit Labeling Based on Optimal Source Domain Selection and Data Synthesis via a Knowledge Graph

**DOI:** 10.34133/plantphenomics.0067

**Published:** 2023-07-27

**Authors:** Wenli Zhang, Yuxin Liu, Chao Zheng, Guoqiang Cui, Wei Guo

**Affiliations:** ^1^ Information Department, Beijing University of Technology, Beijing 100022, China.; ^2^ Graduate School of Agricultural and Life Sciences, The University of Tokyo, Tokyo 188-0002, Japan.

## Abstract

Although deep learning-based fruit detection techniques are becoming popular, they require a large number of labeled datasets to support model training. Moreover, the manual labeling process is time-consuming and labor-intensive. We previously implemented a generative adversarial network-based method to reduce labeling costs. However, it does not consider fitness among more species. Methods of selecting the most suitable source domain dataset based on the fruit datasets of the target domain remain to be investigated. Moreover, current automatic labeling technology still requires manual labeling of the source domain dataset and cannot completely eliminate manual processes. Therefore, an improved EasyDAM_V3 model was proposed in this study as an automatic labeling method for additional classes of fruit. This study proposes both an optimal source domain establishment method based on a multidimensional spatial feature model to select the most suitable source domain, and a high-volume dataset construction method based on transparent background fruit image translation by constructing a knowledge graph of orchard scene hierarchy component synthesis rules. The EasyDAM_V3 model can automatically obtain fruit label information from the dataset, thereby eliminating manual labeling. To test the proposed method, pear was used as the selected optimal source domain, followed by orange, apple, and tomato as the target domain datasets. The results showed that the average precision of annotation reached 90.94%, 89.78%, and 90.84% for the target datasets, respectively. The EasyDAM_V3 model can obtain the optimal source domain in automatic labeling tasks, thus eliminating the manual labeling process and reducing associated costs and labor.

## Introduction

As information and communication technology becomes more prominent, traditional agriculture has gradually incorporated artificial intelligence technology, thereby extending the development direction of intelligent information construction in the fruit industry. Of note, the construction of smart orchards based on deep learning has received considerable attention. High-performance fruit detection technology can be effectively combined with agricultural machinery by extracting various features from images and structured data collected in orchards. This process is appropriate for many smart orchard tasks, such as fruit localization, fruit sorting, fruit yield prediction, and automatic fruit picking [1–4]. Therefore, high-performance fruit-detection technology in orchards is fundamental for the practical establishment of modern smart orchards. Although deep learning-based fruit detection techniques are widely used at this stage, they rely on a large number of labeled datasets to support the training and learning of detection models, thus leading to higher manual labeling costs [5].

Furthermore, because of the currently poor generalization performance of deep learning models, independently producing new fruit datasets and training new detection models for different scenes, environments, shooting methods, and fruit species are time-consuming and labor-intensive. Meanwhile, in actual orchard fruit image collections, fruit trees present dense growth and heavily occluded fruits and branches and most fruits represent small-scale targets. These features increase the difficulty of labeling fruit datasets, thereby increasing the associated time and cost. Therefore, an automatic labeling method that has higher generalizability, stronger domain adaptation, and the ability to satisfy different categories of fruit datasets is urgently required.

In our previous work, we proposed the EasyDAM series, which generates simulated fruit images in the target domain via a generative adversarial network (GAN). Then, the simulated image is input into the fruit detection network as a training set for learning the fruit features of the target domain to obtain the label information. EasyDAM_V1 [6] achieved automatic labeling with datasets that have a similar shape, such as oranges, tomatoes, and apples, and EasyDAM_V2 [7] improved the generation capability of the previous version, which allowed it to be applied to datasets that have different shapes, such as pitaya and mango. Thus, the labor cost required for fruit dataset labeling is reduced. However, 2 problems were observed in the previous studies:

1. The problem of adaptability when more fruit datasets are included within a domain: The process of selecting the source domain datasets for the EasyDAM_V1 and EasyDAM_V2 methods was subjective, and the factors driving the selection of the training set were not considered. This resulted in fluctuating performance when using fruit translation models in different types of agricultural orchards. This unstable performance posed considerable challenges in terms of model construction and applying the method on the ground.
2. The problem of labeling cost of the source domain: Previous EasyDAM methods still required the selection of labeled source domain fruit data or manual labeling of the source domain fruit dataset. These methods relied on at least 1 high-quality dataset of actual orchard scenes in the source domain. Although the method could achieve automatic labeling of fruits in the target domain, it still required considerable human effort to collect and produce the source domain dataset. Thus, manual processes could not be completely eliminated, and the goal of zero-cost automated label generation was not achieved.

Therefore, we performed research related to image translation models and methods to reduce the cost of labeling. Our goal is to develop an objective and quantifiable method to select the optimal source domain fruit datasets that simultaneously correspond to different target domain fruits and design a dataset production method that can automatically obtain labeled data from the training set. Thus, we hope to achieve a zero-cost method for the automatic labeling of fruit datasets.

To address the first problem listed above, this study investigates a method for selecting source and target domain datasets for current image conversion models, among which high-performance image translation models use GAN techniques [8]. Most of the available datasets are public datasets and specific datasets with artificially imposed large differences in color and small differences in shape and texture features. For example, previous studies have used the following datasets: PHOTO-SKETCH dataset [9–12]; face photo-sketch dataset [10,13]; street scene dataset [14–16]; apple-to-orange dataset [17]; horse-to-zebra dataset [17]; CelebFaces Attributes Dataset (CelebA) [18–20]; Radboud Faces Database (RaFD) [21]; and Animal Faces-HQ dataset (AFHQ) [22]. Although the model performance was improved in these studies, the factors underlying the selection of the source domain dataset have been neglected. The above models are generally based on the actual needs of the target domain and selecting the most appropriate source domain dataset. The problem of poor generalization in current image conversion models is further highlighted by the complex scenes, changing environments, and variety of fruits in the agricultural field. Therefore, an objective selection method for source domain datasets is urgently required to meet actual target domain requirements. Such a method could resolve the limited scope of application of image translation models and facilitate the implementation of a single-class source domain dataset that can be simultaneously converted to a multiclass target domain dataset. These capabilities would enable the image translation model to select the appropriate source domain dataset for model training according to the actual target domain and realize the optimum performance for different categories of the target domain fruits.

To address the second problem listed above, this study explored how to reduce the manual annotation cost via deep learning. Researchers have used small-sample training sets to train deep learning models to reduce annotation costs, and this method has been widely used in various fields. Liu et al. [23] proposed a small-sample wavelet transform fault detection method based on GAN and used the wavelet transform for fault detection, which achieved more than 90% fault detection accuracy in a self-harvested electric field dataset. Gao et al. [24] proposed a small-sample gear surface defect detection method based on deep convolutional GAN and lightweight convolutional neural network, and it could classify defective parts in a single context with a classification accuracy of 98.4%. Li et al. [25] proposed a deep learning method for small-sample learning. The training samples were automatically added by a synthetic sample generator to train the target detection and segmentation models in indoor scenes. They achieved an average accuracy of 60% using the public dataset B3DO [26] for indoor scenes. Hu et al. [27] proposed a Gabor-convolutional neural network detection model. By constructing a library of Gabor convolutional kernels for shape and color, an optimal set of Gabor convolutional kernels was obtained by training, thus increasing the feature extraction capability of the network model. Subsequently, the purpose of small-sample detection was achieved, and the average accuracy was 85.19% for the self-built military object dataset MOD VOC. However, this method can only be applied to simpler scenes and tasks owing to the small number of data samples. Applying it to complex scenes or multitarget detection tasks will lead to a loss of accuracy and poor generalization ability. Therefore, this method is not applicable to automatic labeling tasks. In addition, with the development of synthesis technology, researchers have proposed the use of synthetic dataset-based methods to train deep learning models. Abbas et al. [28] applied the GAN method to an image classification task. A conditional generative adversarial network (C-GAN) was used to generate synthetic images of tomato plant leaves for tomato plant pest identification. However, compared to the target detection task, the image classification task cannot obtain location information on the target and thus is simpler in comparison. Therefore, these methods cannot be applied to automatic-labeling tasks. Skovsen et al. [29–31] analyzed the proportion of grass, clover, and weeds in real clover images, and they applied this proportion as a criterion for the synthesis of the clover dataset. The synthesized dataset was similar to the real scene, the cost of manual labeling was reduced, and the dataset was used in subsequent work for clover phenotype data analysis. This method can accurately and automatically obtain the labeling information of different targets and quickly generate a large number of images. However, clover scenes are usually flat and taken from a fixed top view. In comparison, orchard scenes are richer in content and do not have specific requirements for shooting angles, resulting in a greater diversity of images. Creating images with a high degree of realism would be more challenging. Synthetic datasets have limited application potential in agriculture, and the application scenarios are relatively simple. Researchers in other fields have created more realistic synthetic datasets by establishing more complex synthetic rules. German et al. [32] used the unity development platform to create a synthetic dataset of urban streets. The materials of the different model components and their placement were carefully selected to make them more realistic relative to the actual scene. The accuracy of some targets in real scenes exceeded that trained in the real dataset. This method demonstrates the potential of synthetic dataset construction for deep learning tasks. However, although the method conserved manual labeling costs, it was labor-intensive in terms of creating datasets.

The training set production method based on synthetic dataset construction has great potential in other fields. If applied in a complex orchard scenario, this method would be able to completely eliminate the problem of labeling costs for source domain datasets based on the automatic labeling method and improve the model generalizability and label generation quality. Therefore, this paper focuses on how to avoid manual annotation while creating synthetic datasets that have high realism and strong similarity to the target domain images, which would allow deep learning models to extract more effective target features. The EasyDAM_V3 is proposed in this study, which involves automatic generation of label information of the target domain dataset with high accuracy and without any manual annotation. The features of EasyDAM_V3 are as follows.

1. In response to the suitability problem, more fruit datasets are addressed in current research on fruit image translation tasks. In this study, an extensive analysis of different species of fruit was performed. The most suitable source domain fruit dataset was selected for the target domain using a multidimensional spatial feature model. One type of source domain fruit dataset corresponded to multiple types of target domain fruit datasets, which ensured that the fruit image translation model corresponded to different target domain fruits with higher fidelity of translation generalization.
2. To address the problem of manual labeling, this study constructed a knowledge graph of the synthesis rules of orchard scene hierarchy components. A dataset with label information was generated based on transparent background fruit image translation. In addition, the domain difference between them and the target domain scene was further reduced. The fruit detection algorithm based on an anchor-free detector was used for pseudo-label self-learning so that it could simultaneously address fruit targets of different scales and shapes. Thus, the final label generation accuracy was further improved.

## Materials and Methods

**Fig. 1.**
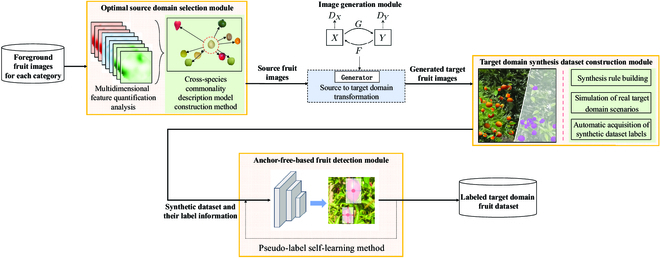
EasyDAM_V3 overall flow chart. The chart includes 3 main contributions (as shown in the yellow rectangle in the figure): (a) Adding a preprocessing algorithm for the optimal source domain selection of fruits before the image generation module to select the most suitable source domain dataset; (b) inputting the fruit image output from the image generation module to the target domain synthesis dataset construction module to synthesize the simulated target domain images using an automated method; and (c) inputting the simulated target domain image to the detection module based on the anchor-free detector to extract the features of the target fruit and background. The label generation quality was further improved using the pseudo-label self-learning method. The final output was the desired fruit label for the actual target domain scene.

The overall proposed scheme for EasyDAM_V3 is illustrated in Fig. 1. The optimal source domain selection module, target domain synthesis dataset construction module, and anchor-free fruit detection-based module in the solid rectangular box with filled lines represent the contributions of this study. This scheme can provide a priori knowledge for use as a guideline for selecting datasets for fruit translation models for target domain data. The most suitable fruit datasets are selected as source domains, and labeling information for a wider range of different categories of fruits is automatically obtained. The contributions of the EasyDAM_V3 method are specified as follows.

To ensure that the image generation model could simultaneously address multiple target domain fruit images using a single source domain fruit image, this study quantified the phenotypic characteristics of different classes of fruits. Thus, the most suitable fruit species for the source domain was selected and was called the optimal source domain fruit. The color, texture, and shape characteristics of the different categories of fruits differed, making it difficult to accurately describe these characteristics with the naked eye. To objectively describe the phenotypic characteristics of fruits, we collected images of 14 fruit datasets belonging 11 species that are widely produced, have large cultivation areas, and present certain research value. Please refer to the contents of Table 1 for details. These were input to the optimal source domain selection module (see Results of target domain fruit generation and synthetic dataset construction). By characterizing different appearance features, different feature descriptions were selected and designed to objectively quantify the phenotypic features of fruit shape, color, and texture. Three feature distance matrices were obtained to represent each of these 3 phenotypic features. The 3 different dimensions of phenotypic features were fused using statistical methods to jointly describe the same fruit characteristic parameters. On this basis, we used the cross-species commonality description model construction method to determine the relative distances of phenotypic characteristics among different fruits. These distances were reconstructed as a spatial fruit feature relative distance map. The datasets of the optimal source domain fruits were selected using a clustering algorithm to obtain the closest average distance to other fruits within the class. This method can obtain the feature distribution space for different species of fruit. In terms of a priori knowledge for the deep learning model, the most suitable source domain fruit dataset was automatically selected according to the types of fruits in the target domain and then input to the fruit translation model for the generation of large-batch target-domain fruit images.

**Table 1.** Species and number of 14 fruit datasets. The sample size of the different datasets of fruits used for phenotypic characterization in this study was 202. In addition, pear_2(white), citrus(orange), apple_2 (red), tomato_1(red), and tomato_2(green) datasets were selected for training the image transformation models in this study. Therefore, additional collection and supplementation of images of the corresponding types of fruits on the internet were carried out.

Species	Datasets	Number of images	Cultivar
Apple	Apple_1(green)	202	Green Apple
	Apple_2 (red)	267	Gala Apple
	Apple_3 (yellow)	202	Banana Apple
Banana	Banana(yellow)	202	Brazilian Banana
Cucumber	Cucumber(green)	202	Fruit Cucumber
Hami melon	Hami melon(green)	202	Xinjiang Hami melon
Kiwifruit	Kiwifruit(brown)	202	Xu Xiang Kiwifruit
Orange	Citrus(orange)	370	Sichuan Citrus
Pear	Pear_1(green)	202	Kulle Balsam Pear
	Pear_2(white)	605	Chinese White Pear
Pitaya	Dragon fruit(red)	202	Red Heart Dragon Fruit
Strawberry	Strawberry(red)	202	Cream Strawberry
Tomato	Tomato_1(red)	283	Ripe Tomato
	Tomato_2(green)	272	Unripe Tomato

As noted above, the previous EasyDAM methods required source domain dataset collection or labeling and could not achieve the automatic labeling of all fruit labels. To effectively reduce the difficulty of image acquisition while also automatically obtaining label information to reduce labor costs, this paper proposed a method to construct a large-batch dataset based on transparent background fruit image translation (see Results of EasyDAM_V3 verification). The fruit synthesis dataset was constructed by establishing a knowledge graph system with the rules for synthesizing the hierarchical components background, leaves, and fruits, thereby simulating a real target domain scene. This dataset was then input to the fruit detection model as a training set to extract the foreground and background features of the fruits in the target domain. This method could automatically obtain various types of label information (e.g., mask labels, polygon labels, and rectangular box labels) of synthetic fruit targets, thereby replacing the manual labeling step and achieving automatic labeling at zero labeling cost.

The synthetic dataset of fruits with labels in the target domain was input to the anchor-free detector-based fruit detection model for initial feature extraction and training of fruit targets. The fruit detection model showed good detection performance when dealing with fruit targets of different sizes in different scenarios. The pseudo-label self-learning method [4] with an adaptive threshold selection strategy was used to cyclically update the pseudo-label of the actual scene fruit dataset in the target domain. Finally, the pseudo-label information of the actual scene fruit dataset of the target domain was output. Thus, the labeling of the actual fruit dataset of the target domain with zero annotation cost was automatically generated.

### Fruit datasets

Two fruit datasets were used in this study: a transparent background fruit dataset and an actual orchard dataset in the target domain. The transparent background fruit dataset was used to describe the fruit phenotypic characteristics in the optimal source domain selection module and generate foreground fruit images in the simulated target domain in the image generation module. The target domain actual orchard dataset was used to provide background images for the target domain synthetic dataset construction module and evaluate the quality of label generation.

#### Transparent background fruit dataset

To more realistically and accurately describe the phenotypic characteristics of different fruit datasets and reduce the operational difficulty of synthesizing images, this study used only the foreground fruit images as the dataset, which contains 14 common fruit datasets available on the market. The resolution was 256 × 256 to allow for feature analysis and deep learning model training. The images in the dataset were obtained from the public dataset fruit360 [33], web crawler, internet collection (without copyright restrictions) and 3-dimensional (3D) scan reconstruction methods to construct transparent background fruit datasets, respectively. Therefore, the transparent dataset does not require manual labeling in the acquisition process. The categories and specific numbers of fruit images are listed in Table 1.

#### Actual orchard fruit dataset

**Fig. 2.**
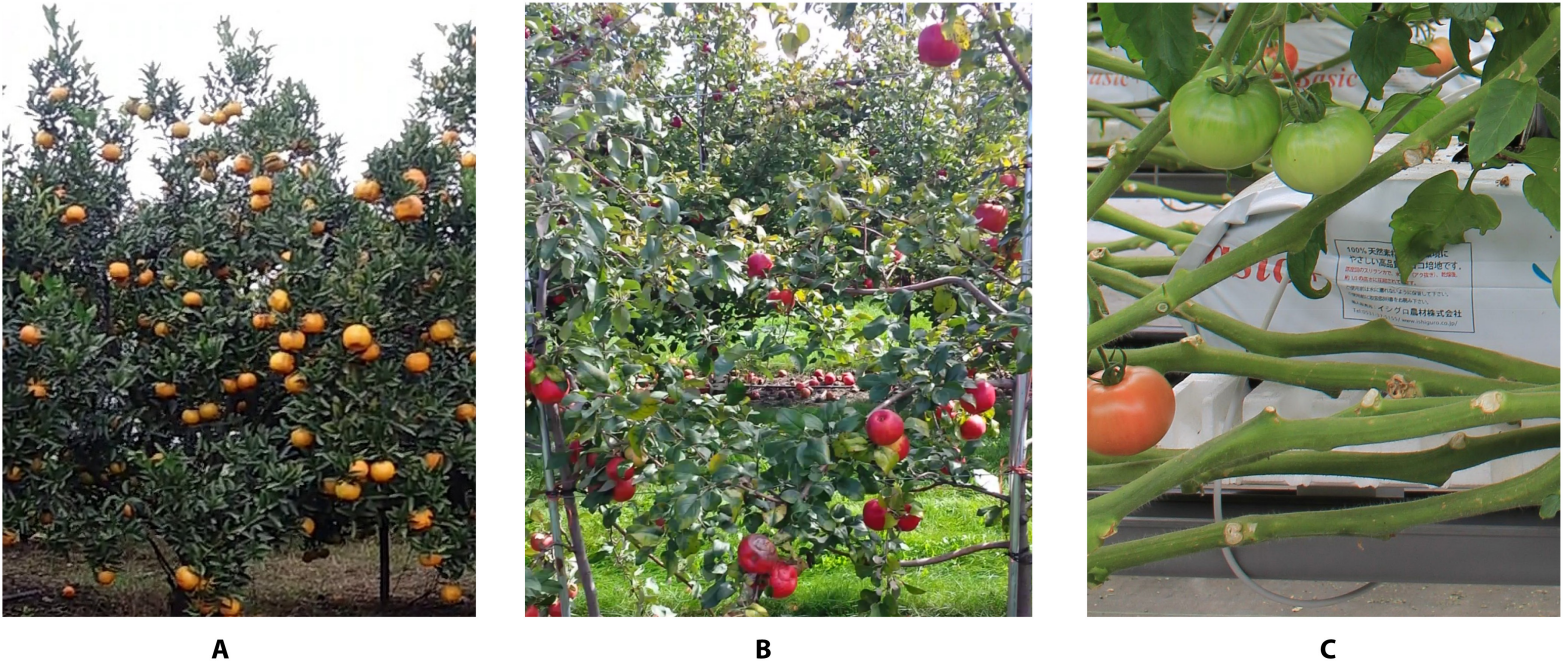
Images of the actual orchard scene in the target domain. (A) Target domain citrus image, (B) target domain apple image, and (C) target domain tomato image.

In this paper, fruit images in actual orchard scenes were also collected and labeled as the target domain test data. This dataset mainly contains 3 types of fruit, citrus, tomato, and apple, as shown in Fig. 2. The specific descriptions are as follows.

1. Citrus dataset: This dataset followed the images from the source domain fruit dataset part of a previous EasyDAM study [4]. The citrus images in this part were mainly collected from a citrus orchard in Sichuan Province, China, which species is Sichuan citrus. A DJI Osmo action camera (Shenzhen DJI Technology Co., Ltd., China) was used as the image acquisition device. A total of 664 citrus images were acquired, including images from various complex scenes such as shaded and backlit images. Among these, 550 images without label information were used as the training set for the experiments. A total of 114 images containing rectangular box label information were used as the experimental test set.
2. Apple dataset: This dataset was obtained from the MineApple dataset [34], which contains images of Gala apples in various highly cluttered environments. A total of 504 unlabeled fruit images were selected as the experimental training set, and 82 labeled fruit images were used as the experimental test set. In addition, we cropped the test set images to eliminate the effect of apples falling on the ground on the final detection results.
3. Tomato dataset: This dataset was derived mainly from the dataset published by Mu et al. [35]. The dataset was collected from 2 farms in Tokyo, Japan, and contains ripe red tomatoes and unripe red tomatoes, with unripe green tomatoes accounting for the largest proportion. Among these, 598 images without label information were used as the training set for the experiments. A total of 102 images were used as the experimental test sets.

### Optimal source domain selection module

**Fig. 3.**
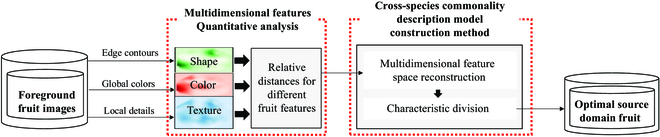
Flow chart of the optimal source domain selection method for fruit generation corresponding to multiclass target domains.

The optimal source domain selection module for the target domain was used to describe and analyze the phenotypic characteristics of different classes of fruits. The flow of this module is shown in Fig. 3. This module provides guidelines for the selection of datasets and the setting of training parameters for deep learning by calculating the commonality among their features as priori knowledge for deep learning algorithms. This module contains 2 main parts. First, a multidimensional feature quantification analysis method is proposed to analyze and describe the appearance characteristics of different fruit individuals. Second, a cross-species commonality description model is constructed to classify different fruits according to their phenotypic characteristics and select the optimal source domain fruit datasets from them.

#### Multidimensional feature quantification analysis

**Fig. 4.**
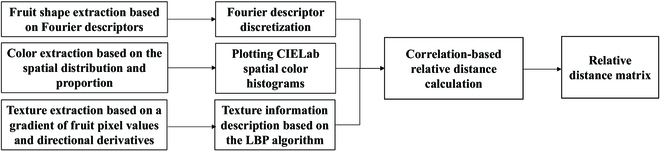
Multidimensional feature quantification analysis chart.

Quantitative descriptions of the phenotypic features of crops provide important indicator information for evaluating the crops’ characteristics. This method can help researchers in fields outside of agriculture, such as in animal husbandry—by improving the breeding efficiency and increasing yield—and in computer vision—by improving the training and feature extraction by deep learning networks. Among these indicators, the color, shape, and texture features of crops are important indicators of their appearance, which can quantify the appearance of crops in an objective, differentiated, accurate, and direct manner [36,37]. In the field of image translation, researchers have focused more on improving models. In contrast, the quantitative description of fruit features in the construction of smart agriculture can be applied as priori knowledge for the selection of datasets and adjustment of training parameters for image translation models, which improves the generalizability of the models. Therefore, this paper proposed a multidimensional feature quantification description method focusing on the appearance of different categories of fruits to calculate the magnitude of their feature differences. The flow of this method is shown in Fig. 4.

In this study, we characterized 14 fruits in 3 dimensions, including shape, color, and texture. To ensure the universality of the method, we chose methods that were rotation invariant, translation invariant, scale scaling invariant, and consistent in describing the subdimensions. Subsequently, we formed a high-latitude feature distance matrix by calculating the feature differences between the 2 fruits, and the relative distances were used to represent fruit features.

Shape characteristics: In this paper, Fourier descriptors were used to construct the boundary profile curve of the fruit target into a 1-dimensional sequence. The Fourier coefficients were obtained by performing a discrete Fourier transform on the sequence. The coordinates of the boundary points were used to describe the shape of the boundary against the sequence coefficients. We supposed that there were *N* boundary points on the boundary of the fruiting target with the starting point ( *x*_0_, *y*_0_ ). The boundary could be represented as a sequence of coordinates *s*(*k*) following the inverse direction.

$$s\left(k\right)=\left[x_{k},y_{k}\right],\;k=0,1,2,\ldots \;\ldots ,N-1$$ (1)

To change the 2-dimensional (2D) coordinates into a 1-dimensional sequence, we introduced a complex representation method. We considered the target boundary as a complex function starting from a certain point and rotating the perimeter of 1 week counterclockwise along the boundary. The sequence of coordinates *s*(*k*) was expressed as follows:

$$s\left(k\right)=x_{k}+j*y_{k},\;k=0,1,2,\ldots \;\ldots ,N-1$$ (2)

The complex *s*(*k*) sequence is denoted by the 1-dimensional discrete Fourier transform coefficients *a* (*u*):

$$a\left(u\right)=\frac{1}{N}\left(\sum_{k=0}^{N-1}s\left(k\right)e^{-j2\prod \frac{\mathrm{ku}}{N}}\right),u=0,1,2,\ldots ,N-1$$ (3)

where *a*(*u*) denotes the Fourier descriptor, which was used to represent the results of the frequency analysis of the boundary contour of the fruit target. Subsequently, we normalized the Fourier descriptors so that they had translation, rotation, and scale invariance and were thus unaffected by the position of the contour in the image, the angle, or the scaling of the contour. These descriptors are widely used in real-world scenarios. The normalized Fourier descriptor *d*(*u*) can be expressed as follows:

$$d\left(u\right)=\frac{||a\left(u\right)||}{||a\left(0\right)||},\;u=0,1,2,\ldots \;\ldots ,N-1$$ (4)

The shape difference between different fruit individuals was then calculated based on the Pearson correlation coefficient:

$$\begin{array}{c} \rho \left(i,j\right)=\\ \frac{E\left(d{\left(u\right)}_{i}\cdot d{\left(u\right)}_{j}\right)-E\left(d{\left(u\right)}_{i}\right)\cdot E\left(d{\left(u\right)}_{j}\right)}{\sqrt{E\left(d^{2}{\left(u\right)}_{i}\right)-E^{2}\left(d{\left(u\right)}_{i}\right)}\cdot \sqrt{E\left(d^{2}{\left(u\right)}_{j}\right)-E^{2}\left(d{\left(u\right)}_{j}\right)}} \end{array}$$ (5)

where *i* and *j* represent 2 different fruit targets. Because the energy of the shape is mostly concentrated in the low-frequency region, the high-frequency component of the Fourier transform is generally small and easily disturbed by high-frequency noise. In general, only the low-frequency component of the normalized descriptor is used to describe the shape of the fruit target (*u* = 16 based on experience). At this point, the Fourier descriptor has the best discrimination between different fruits, that is, the largest difference in the shape of the object.

Color characteristics: To extract quantitative color distribution information, this study used a method to convert the global color information of fruits from the RGB color space to the CIELAB color space, enabling the computer to recognize and process colors in a continuous color space. Then, the pixel means and variances of the L, a, and b values of each fruit were calculated and normalized. Histograms were drawn to represent the distribution of color patterns for all fruits. The color descriptors were calculated as follows:

1. The number of pixels falling into each interval is calculated as *N_i_*, where *i* is the number of intervals. In this study, every 20 points were divided into 1 interval according to the range of L, a, and b values.
2. The percentage of the number of pixels in each interval is calculated as follows:
  $${\text{Rate}}_{i}=\frac{N_{i}}{N_{\mathrm{all}}}$$ (6)

where *N_all_* denotes the total number of pixel points in the foreground area of the fruit.

The mean value of each interval pixel point’s L, a, and b values is calculated as *Mean_i_*.

3. The product of the percentage of pixels in each interval and its mean value is calculated as follows:
  $$\mathrm{LAB}\left(x\right)=\sum_{i=1}^{n}{\text{Rate}}_{i}\times {\text{Mean}}_{i}$$ (7)

where *LAB*(*x*) denotes the pixel mean and variance of the L, a, and b values.

4. The differences in color characteristics *D_c_* between the different fruit targets using the L2 distance are calculated as follows:
  $$D_{c}=\sqrt{{\left(L_{i}-L_{j}\right)}^{2}+{\left(a_{i}-a_{j}\right)}^{2}+{\left(b_{i}-b_{j}\right)}^{2}}$$ (8)

where *L_i_*, *L_j_*, *a_i_*, *a_j_*, *b_i_*, *and b_j_*, denote L, a, and b values of 2 different kinds of fruits i and j respectively.

**Fig. 5.**
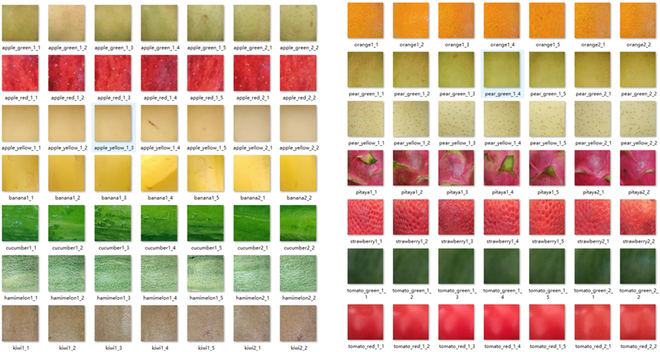
Local texture blocks of the fruit target obtained by random sampling selection.

Texture characteristics: Texture features do not depend on color or brightness but reflect important information about the organization of surface structures and their variation of the values of the surrounding pixels. Owing to the characteristics of foreground fruit images, including uneven illumination, severe edge contrast, and texture heterogeneity, there is a large error in directly characterizing the foreground global image. Therefore, this study used random sampling to randomly select *N* local texture blocks in each foreground fruit image as the input, as shown in Fig. 5. The gradient and directional derivative information among their pixel points were then calculated using a local binary pattern (LBP) descriptor [38]. Textural features were then extracted and normalized. LBP texture descriptors are guaranteed to be rotation translation invariant and scale scaling invariant. Subsequently, the Pearson correlation coefficient shown in Eq. 5 was used to calculate the differences in texture features among the different fruit targets.

#### Cross-species commonality description model construction method

For the 14 datasets of fruit samples selected in this study, 3 different feature dimensions were selected for each fruit. The relative distances between the 3D fruit appearance features were obtained using image analysis methods. Because of the large number of fruit types and phenotypic features, the dimensionality of this description method is high, and it is difficult to intuitively obtain the optimal source domain directly based on the relative distance of a single feature between the 2 fruits. Therefore, this study proposed a method for constructing a cross-species commonality description model. A multidimensional feature space was constructed based on the relative distance between 2 fruit features. The objective was to convert the relative distances between different fruit features into absolute distances in the same feature space. The phenotypic characteristics of each fruit image could be concisely and precisely described using a pair of 2D planes of position coordinates. Subsequently, the feature points in the space were divided using a clustering algorithm, and clustering was performed according to the similarity of the fruit features. Thus, the optimal source domain fruits in the different clusters were obtained. The cross-species commonality description model was constructed as follows:

1. Because different distance descriptions were used to calculate the shape, color, and texture features in this study, the distance quantization numerical scales of the different features were not uniform. Therefore, it was necessary to normalize the scale of the 3 feature descriptors. It is convenient to construct the feature space afterwards. The scale normalization method is given by the following equation:

$${\text{scale}}_{\text{feature}}=\frac{\max\left\{\left[\max\left(x_{s}\right)-\min\left(x_{s}\right)\right],\left[\max\left(x_{c}\right)-\min\left(x_{c}\right)\right],\left[\max\left(x_{t}\right)-\min\left(x_{t}\right)\right]\right\}}{\max\left(x_{\text{feature}}\right)-\min\left(x_{\text{feature}}\right)}$$ (9)

where *x_s_*, *x_c_*, *x_t_* denote the relative distances of the shape, color, and texture, respectively, between 2 fruit images; *scale_feature_* denotes a scale-normalized scale. Because each phenotypic feature is to be scaled separately, and *feature* represents the shape (s), color (c), and texture (t) in sequence.

2. Because the distance feature matrix can only represent the relative distances of features between fruit images, which is not sufficiently intuitive, the multidimensional scale analysis algorithm was used in this study. The algorithm uses the principle of constant relative distance between 2 points; thus, the multidimensional distance matrix is visualized in a 2D plane coordinate system for different phenotypic features of the fruit. Three different 2D coordinate systems with absolute distances of fruit features are then constructed based on these 3 phenotypic features. By extracting the position coordinates of fruit images in different coordinate systems, phenotypic features are combined using a principal component analysis dimensionality reduction algorithm, and these features are jointly represented in a 2D planar space. The reduced dimensional shape features are used as the x-axis, and the reduced dimensional color and texture features are used as the y-axis. A new multidimensional feature space with 3 fused phenotypic features is constructed, and the fruit image distribution is obtained.
3. To obtain the optimal source domains of the14 fruit datasets through the constructed multidimensional feature space analysis, this study used a clustering algorithm to classify the fruit location situation in the space. Because the manual determination of the optimal number of source domains is subject to certain errors and the processing is not sufficiently objective, the density-based spatial clustering algorithm was used in this study. This algorithm is used to automatically classify and select the fruit datasets of the source domains and the number of source domains. The number of clusters is automatically determined according to the distribution of differences in fruit characteristics. The fruit datasets at the geometric center within each cluster are used as the optimal source domain fruit datasets.

Using the above method, the degree of similarity of phenotypic feature differences between different fruits can be obtained visually. Thus, the optimal source domain fruit datasets that simultaneously correspond to different target domain datasets can be selected scientifically and objectively. This information can be used as priori knowledge for the subsequent image-translation model, and it may represent a guideline to generate more realistic target domain fruits and multispecies target domain fruit images from a single source domain dataset. Thus, the stability of the fruit translation model training is improved.

### Target domain synthesis dataset construction module

During the construction of synthetic orchard images, obtaining a more realistic and detailed synthetic dataset by relying entirely on the method of randomly placing background-free fruit images in the background is difficult because of the complex orchard scenes and changing environments. It also cannot help the fruit detection model extract the fruit features in the target domain better.

**Fig. 6.**
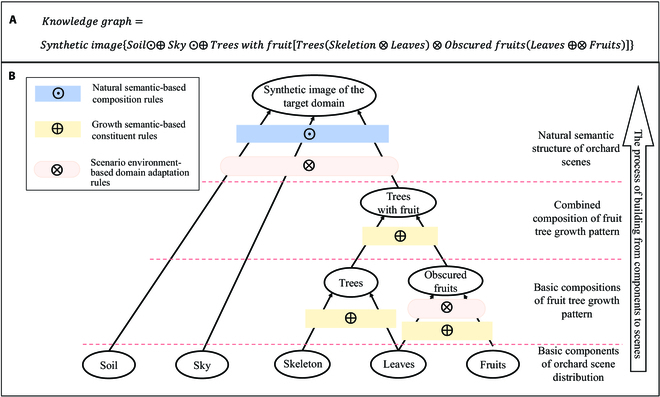
Knowledge graph system based on the synthesis rules of hierarchical components. (A) Containment relationships between elements of different levels and their composition rule expressions. (B) Knowledge graph architecture diagram of the orchard scene.

To make the constructed synthetic dataset approximate the real target domain fruit growth scene and posture and train the fruit detection model effectively, this study analyzed the structural and regular relationships of different elements in real orchard scenes in detail. A knowledge graph system based on the synthesis rules of hierarchical components was established using the distribution rules of semantic structures in real scenes as shown in Fig. 6. The figure clearly shows that this study follows the logical relationship from the lower-level components to the upper-level scenes for the hierarchical construction of the knowledge graph, which contains 4 levels and 3 rules. The 4 levels from lower to upper are the basic components of orchard scene distribution, the basic compositions of fruit tree growth patterns, the combined composition of fruit tree growth patterns, and the natural semantic structure of orchard scenes. The 3 rules are different construction guidelines in the synthetic dataset: natural semantic-based composition rules, growth semantic-based constituent rules, and scenario environment-based domain adaptation rules. Each layer of the knowledge graph contains unique elements. The upper-level elements are constructed using the lower-level elements according to the above 3 rules.

The natural semantic-based composition rules are used to indicate that the overall construction of the synthetic image will follow the natural growth of the plants in the orchard and the scene distribution of the whole orchard scene image consisting of sky-ground-tree. These rules can help the computer vision field by providing a better understanding of images from a global scale. Since the fruits and trees in the image are affected by growth changes and scene environment changes, this paper extends 2 rules based on local semantics from natural semantics, namely, growth semantic-based constituent rules and the scenario environment-based domain adaptation rules. Among them, the scenario environment-based domain adaptation rules are used to make the synthesized image closer to the real target domain scene. It is possible to standardize the synthesized image of obscured fruits as well as the synthesized image of the target domain. The growth semantic-based constituent rules are used to normalize the synthesis of the base constituent as well as the combined constituent. The rules analyze the growth direction of trees and fruits from the foreground and background parts, respectively, simulating the real fruit tree growth dynamics.

To better implement the construction of synthetic datasets, a hierarchical structure relationship in the knowledge graph was constructed in this study. The 3 rules were divided more carefully according to the different hierarchical levels. The hierarchical structure relationship of the knowledge graph can be expressed as follows:
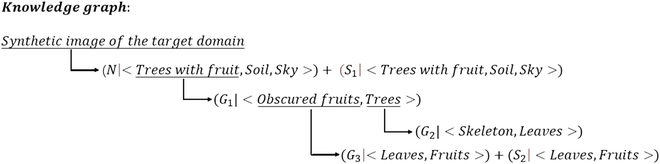


where *N* denotes a composition rule based on natural semantics; *S* denotes the scenario environment-based domain adaptation rules that contain 2 subrules; *S*_1_ denotes the background domain adaptation subrule; *S*_2_ denotes the light and size subrule; *G* denotes the growth semantic-based constituent rules that contain 3 subrules; *G*_1_ denotes the fruit growth subrule; *G*_2_ denotes the branch and leaf colonization subrules; and *G*_3_ denotes the graded shading subrules. The pseudocode implementation of the construction method for the synthetic dataset is presented in Algorithm 1. The specific description of each of these rules is as follows.

Natural semantic-based composition rules: The scenario environment and growth state of fruits in real orchards follow certain patterns. If the method of randomly placing foreground fruit images in background images is adopted directly, it does not conform to the basic composition rules of orchard scenes and cannot build a high-quality synthetic dataset. Therefore, in this study, the construction of a synthetic dataset was normalized using natural semantic-based composition rules. In addition to following the spatial distribution rules for the sky, fruits, trees, and land from top to bottom, this method restricts the growth area of fruits. The background image is preprocessed by a threshold segmentation algorithm to distinguish the land, tree, and sky components and mark the area of the tree parts. Subsequently, when placing the fruit target, it is restricted to the tree area, thus generating a more realistic fruit image. In addition, as rules are constructed from the global semantic perspective, the composition rules based on natural semantics include 2 types of rules, namely, growth semantics and scenario environment, which will be introduced in this paper.

Growth semantic-based constituent rules: Fruit growth in orchards is densely distributed and often involves a large number of heavily stacked fruit targets. This type of fruit is obscured by leaves or other fruits, thus causing variations in the semantic information of fruit growth. If they cannot be processed in a reasonable manner, they will cause serious misdetection and misdetection problems. Therefore, in this study, we designed the fruit growth subrule *G*_1_ in the process of forming a tree with fruit trees growing with obscured fruit and simulated the fruit growth dynamics in different kinds of fruit trees to obtain more realistic fruit distribution characteristics. Thus, the fruit tree components that conformed to the growth semantics were obtained. The branch and leaf colonization subrule *G*_2_ was designed in the process of tree formation by skeleton and leaves; therefore, the leaves were dynamically adjusted according to the growth direction of the branches and leaves. Leaves were distributed in the tree skeleton according to a certain growth pattern. In the process of forming obscured fruits from leaves and fruits, the graded obscuration subrule *G*_3_ was designed. By setting the obscuration ratio discriminant module between leaves and fruits and between fruits and fruits, the obscuration ratio of fruit targets in the image is randomly distributed in the interval [0, 0.5].

Scenario environment-based domain adaptation rules: Because of the different growth environments of fruits in different orchards, shooting angles, shooting distances, and other human collection factors are subject to change. The fruit in the actual scenario is affected by external lighting, shading, and growth factors, which can lead to large differences in their color characteristics. The transparent background fruit images used in this study were obtained by translating the fruit images from a single-source domain. The variability in the orchard scenes was not considered. Relying only on the background and leaf images of the source domain in the previous research method as components to construct the synthetic dataset leads to domain adaptation of the training model in different target domain scenes, which is not guaranteed. Therefore, in this paper, the background domain adaptation subrule *S*_1_ was proposed for the process of forming synthetic images of the target domain by land, sky, and trees with fruits. The background images in the real orchard of the target domain were used as the background images in the synthetic dataset. This enables the detection model to learn the feature information of the target domain foreground as well as the background. The illumination and size subrule *S*_2_ was designed to form obscured fruits with leaves. The foreground fruit images were randomized in terms of saturation, brightness, and rotation angle to increase the diversity of fruit samples.


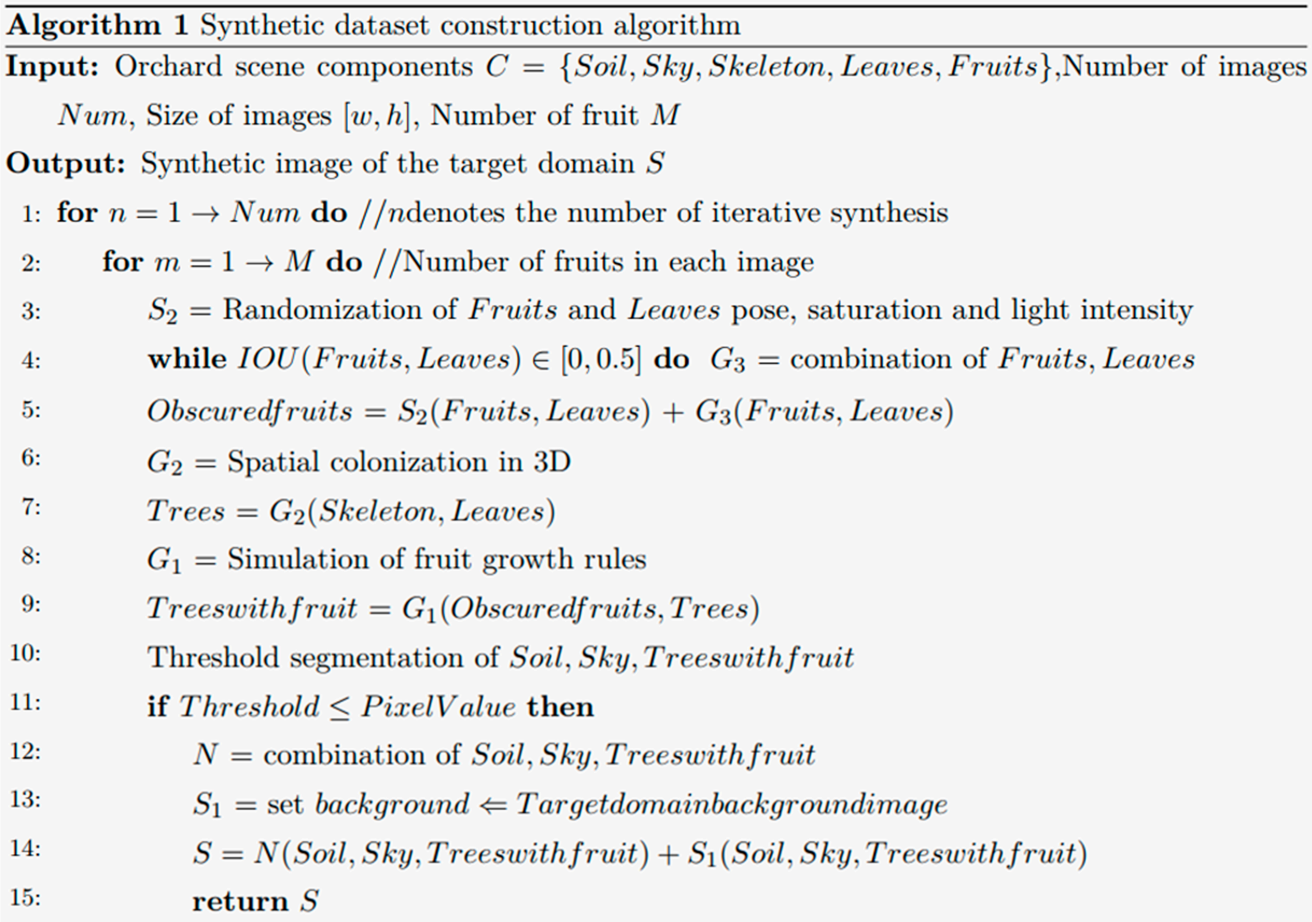


We dynamically adjusted the size of the fruit to adapt to the location and angle of the camera shot according to the specific application scenario. The dataset obtained using this method was sufficient to support the training of detection models and feature extraction as well as a certain degree of domain adaptation. The accuracy of the target domain fruit label generation in different target domain scenes was significantly improved. The components used in the process of constructing the synthetic dataset have their own category information and original scale information. The location of placement and the scaled scale information can be recorded during the placement process. The synthetic dataset algorithm can automatically construct labels from the above information. Therefore, the process is automatic and does not require manual labeling. We hope that this knowledge graph system will provide a proposal and idea for constructing a dataset for orchard scenes. Because this study adopts a completely automated method to place fruit targets, the labeling information of fruit locations in the synthetic dataset can be obtained automatically. Thus, automatic fruit labeling in the target domain with zero labeling cost was achieved.

### Anchor-free-based fruit detection module

In this study, a target domain dataset simulated by the CenterNet detection algorithm [39] based on an anchor-free detector was used for training. This method can detect the target as a point and predict its offset using the extracted features to determine its specific location. Thus, it eliminates the dependence of anchor-based algorithms on the manual setting of prior bounding boxes, such as the YOLO series [40,41]. On this basis, the pseudo-labels generated by the CenterNet detection algorithm were then updated recurrently using the pseudo-label self-learning method with adaptive thresholds [4]. The number and scoring information characteristics of the generated pseudo-labels under the corresponding confidence thresholds were calculated to obtain the quality variance values of the pseudo-labels. Then, the confidence threshold corresponding to the maximum value of the variance was used as the confidence threshold balance point (i.e., the optimal confidence threshold) for the pseudo-labels. Pseudo-labels can be divided into 2 categories, high-quality labels and low-quality labels, and their differentiation is maximized. In the next step, high-quality species pseudo-labels were selected, the detection model was trained, and the confidence threshold was adjusted dynamically. Thus, the generated pseudo-labels were updated, and the performance of the fruit detection model and accuracy of the generated pseudo-labels were gradually improved.

### Experimental strategy and evaluation metrics

The training strategy used in this paper for deep learning model training is as follows. The CycleGAN translation model mainly used in the experiments of this paper is unified with the following hyperparameters during the training process: A small-batch adaptive moment estimation optimizer (Adam) with a momentum factor was used to optimize the network, where the Momentum factor Momentum value was set to 0.5. The batch size was set to 1. The learning rate was set to 0.0002 for the first 150 training rounds. The learning rate was reduced to 0 in the second 150 training rounds with linear decline. The training device we used was an NVIDIA Geforce GTX 3090, and the time spent for each model training was 3 h. The CenterNet detection model mainly used in this paper is set to have a batch size of 4 and an epoch of 100, and the rest of the hyperparameters are the same as the original model. The training device we used is NVIDIA Geforce GTX 3090, and the time spent for each model training is 1 h.

Because the final task of the proposed method was to automatically generate target location labels in different scenes, we chose to use the detection performance of the detection model in target domain scenes as an evaluation index. The effectiveness of the method proposed in this study was indirectly verified by measuring whether its prediction frame was sufficiently accurate. The 3 main metrics used were precision, recall, and average precision. A higher value of the corresponding metric indicated a higher quality of label generation in the target domain scenario. Additionally, the precision and recall rates used in this study were obtained from the values at the balance point.

## Results

We selected 3 representative fruits (citrus, apple, and tomato) as the target domains. The intraclass clustering center fruits were used as the source domain fruits for fruit transformation and label generation experiments. The experiments were divided into 3 main parts.

1. We provided a multidimensional feature quantization description as well as spatial reconstruction using transparent background fruit images and selection to obtain the optimal source domain fruit (Results of optimal source domain selection).
2. The optimal source domain fruits were input into the image translation model CycleGAN. The foreground fruit images of the target domain were trained and generated. We then used it to simulate the construction of synthetic datasets of citrus, apple, and tomato scenes in the target domain (Results of target domain fruit generation and synthetic dataset construction).
3. The above synthetic datasets were input into the anchor-free detector-based fruit detection model for training. The self-learning method of pseudo-labeling based on threshold segmentation proposed in a previous study was used to obtain pseudo-labeling of the unlabeled target domain citrus, apple, and tomato datasets. The 3 experiments were denoted as pear2orange, pear2apple, and pear2tomato (Results of EasyDAM_V3 verification).

### Results of optimal source domain selection

**Fig. 7.**
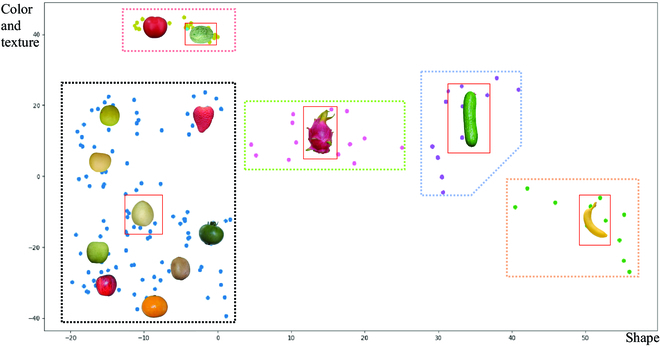
Scatter plot of the multidimensional feature space reconstruction and optimal source domain selection results. Each rectangular dashed box of a different color represents a different cluster. The fruit type at the center of each cluster is highlighted with a solid red line box, representing the optimal source domain fruit datasets in that cluster, where the x-axis represents the distribution of fruit shape features. As the color and texture features of fruits in the actual scene are influenced by the environmental lighting, shooting angle, and other factors, the color and texture features were jointly represented as the y-axis of the multidimensional feature space in this study.

In this section, we analyzed the 3 phenotypic features shape, color, and texture for images of 14 different fruit datasets. Subsequently, a multidimensional feature space reconstruction was performed. A clustering algorithm was used to cluster and classify fruits to select the optimal source domain fruit datasets. Figure 7 shows the results of the multidimensional feature space reconstruction and distribution of the 14 classes of fruits. We analyzed and discussed the results of the multidimensional feature space reconstruction map presented in Fig. 7.

1. Citrus(orange), apple_2(red), and tomato_2(green) fruits were all in the same cluster. The optimal source domain for these 3 target domains was pear_2(white). Therefore, the pear_2(white) was selected as the optimal source domain for subsequent experiments.
2. For other fruit datasets, most of the results in the multidimensional feature space distribution figure were consistent with what one would expect from intuitive observations, such as the close proximity of citrus to apple, that is, the features of these 2 types of fruits were relatively similar. This also validates the effectiveness of the CycleGAN paper using the Apple to Orange (A↔O) dataset for training.
3. In addition, most fruits were distributed in the lower-left region. The center of its clustering was pear_2(white), indicating that the average distance through pear_2(white) was closer to the fruits of the other 8 datasets. More realistic results could be achieved for the 8 datasets of fruits when using only the pear_2(white) dataset for image translation. The cost of the image collection task was significantly reduced, and the applicability of the automatic annotation algorithm was improved.
4. The features of hami melon(green) and tomatoe_1(red) were not expected to be very similar primarily because the texture and smoothness of fruit in 2D images are difficult to describe. However, the textural features of these 2 types of fruit were similar (see Fig. S4 in Supplementary Materials for details). Therefore, in the image translation task between 2D images, hami melon(green) was more easily converted to the image of tomato_1(red).
5. The 3 fruits pitaya(red), cucumber(green), and banana(yellow) were diverse owing to their own phenotypic characteristics. For example, cucumber and banana have straight and curved forms, while pitaya has a nonuniformly distribution of surface scales. Therefore, the fruit distribution is more discrete and constitutes a separate species. For image translation, fruits in nearby clusters, such as strawberries and green tomatoes, could be selected.
  Subsequently, we used the results as priori knowledge for the image translation model to guide the image translation model in selecting the most appropriate source domain target domain dataset for training and learning. In addition, we only statistically described the phenotypic characteristics of 14 fruit datasets due to labor costs, seasonal constraints, and other factors. However, we hope that the optimal source domain selection process proposed in this study can be used as a general description method for target features. This process is not limited to the 14 fruit datasets but rather can further expand the selection range for fruit, or even other objects, in the characterization task. To more intuitively introduce and demonstrate the methods in each part of this paper, we present detailed intermediate results obtained in each step, including the visualization results after the extraction of features by texture feature descriptors (as shown in Supplementary Materials, Fig. S1), the results of 3 single-feature space reconstructions of shape color and texture (as shown in Supplementary Materials, Figs. S2 to S4), and the different samples of 14 fruits in the multidimensional feature space reconstruction map positions (as shown in Supplementary Materials, Fig. S5).

### Results of target domain fruit generation and synthetic dataset construction

**Fig. 8.**
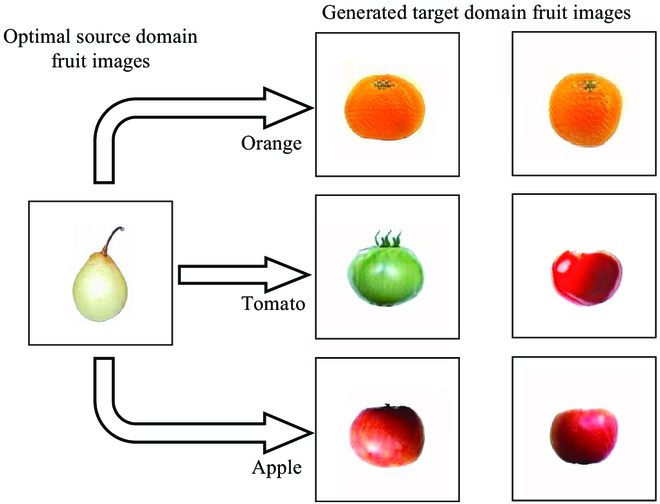
Visualization results of target domain fruit image generation using the CycleGAN model.

Figure 8 shows that the 3 target domains of citrus, apple, and tomato are concentrated in the lower-left region, which is rich in experimental samples. Therefore, we selected fruits from this cluster for further experiments. We verified the reasonableness of the optimal source domain selection results. The selected target domain fruits were citrus, apple, and tomato, while the source domain dataset was pear_2(white), according to the calculation results. In this section, the foreground images of the source domain of pear_2(white) were translated to citrus, apple, and tomato foreground images using the image translation model CycleGAN. The generated images were then input to the target domain synthetic dataset building module to obtain the synthetic fruit dataset as the training set for the detection model. The image-translation part is presented in Target domain fruit image translation. The synthetic dataset is presented in Synthetic dataset generation.

#### Target domain fruit image translation

Most image-translation models are unsupervised learning methods. A limitation of such methods is that they cannot accurately distinguish the foreground from the background, which is why the image translation model in the current study can only play the role of target recoloring and cannot produce large deformations in the target. Based on this, we used the CycleGAN model to translate a pear_2(white) image with a transparent background into citrus, apple, and tomato images in the target domain. The phenotypic features of the fruit images with a transparent background were obvious. Errors are minimized when analyzing fruit features with quantization, and fruit images with a transparent background have relatively simple information, which makes it easier for the image translation model to learn the effective features. Thus, more realistic fruit images were generated in the target domain, which is consistent with the requirements of our study.

In this study, the CycleGAN image translation model was trained using 403 foreground fruit images of pears. A total of 403 images of citrus, apple, and tomato were obtained separately. To better match the fruit characteristics in an actual tomato orchard, we conducted 2 experiments on the transformed part of the tomato images to obtain both red and green tomato images. Figure 8 shows the visualization results of translating pear images into citrus, apple, and tomato images using CycleGAN. The fruit images produced large feature changes during the translation process, which led to more realistic target domain fruit images while also strengthening the fruit diversity. Subsequent training of the fruit detection model will allow more and richer features to be learned from fewer datasets, thus further improving detection performance.

Because this study used a fruit dataset with a transparent background, the target features in the images were obvious, which could significantly reduce the performance requirements of image translation models. Most mainstream image-translation models at this stage can accomplish this task well. Therefore, using our model provides a great deal of flexibility in the dataset selection for automatic annotation and can eliminate the need and dependence of image translation models on ultrahigh-performance graphics processing unit and other graphic processing devices.

#### Synthetic dataset generation

In this study, regions without fruits in the actual orchard dataset in the target domain were cropped and stitched. Ten leaf images were obtained as components to simulate an occlusion scene. The foreground target domain fruit images obtained from the CycleGAN output were then used to obtain 1,000 synthetic datasets each for citrus, apple, and tomato, which were used as the training set for the fruit detection model using the target domain synthetic dataset construction method. The trained models could be used as pretrained models in the subsequent pseudo-labeling self-learning method, and they were denoted as $M_{\text{orange}}^{1},M_{\text{apple}}^{1},M_{\text{tomato}}^{1}$.

**Fig. 9.**
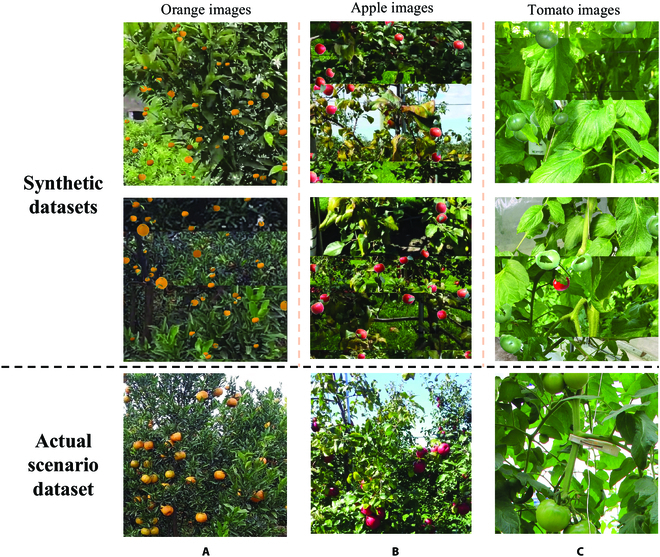
Synthetic dataset images. Synthetic images of (A) citrus, (B) apple, and (C) tomato are shown.

The synthetic dataset obtained by the target domain synthetic dataset construction method is compared with a real image in Fig. 9. The synthetic dataset approach is used to simulate the shooting angle, scene complexity, target fruit size, and lighting variations of the actual target domain dataset. The synthetic dataset approach enables the fruit detection model to learn effective target domain features for better automatic fruit labeling tasks. In addition, it has high domain adaptation in practical scenarios. Therefore, this dataset can be used to fully train the fruit detection model to achieve higher label generation accuracy, and it can also further reduce labor costs. This solves the problem where the EasyDAM method still needs to annotate the source domain dataset, which cannot be used for manual labeling. Thus, cross-species fruit detection with zero labeling and automatic label generation can be achieved.

### Results of EasyDAM_V3 verification

In the experimental work described in this section, we pretrained the CenterNet detection model for the 3 target domain datasets citrus, apple, and tomato using a synthetic fruit dataset based on the target domain. In addition, the label frame information is optimized using a pseudo-label adaptive threshold selection strategy. The trained models *M_orange_*, *M_apple_*, *M_tomato_* are input to the real orchard images for testing the detection accuracy.

In addition, the comparison algorithm uses the automatic labeling method EasyDAM series methods in the previous study. Among them, the experimental objects of EasyDAM_V1 and EasyDAM_V2 are different. Moreover, EasyDAM_V1 is mainly oriented to the task of automatic fruit labeling with similar phenotypic features. EasyDAM_V2 is mainly for the automatic labeling task when there are certain differences in phenotypic characteristics, and this paper focuses on the selection of the optimal source domain as well as label generation experiments for fruits with similar features in the same cluster. In addition, EasyDAM_V1 achieves the SOTA results so far in the task of automatic labeling of datasets with similar phenotypic features. Therefore, in this paper, EasyDAM_V1 is used as the comparison algorithm for the orchard scene dataset (denoted as EasyDAM dataset) generated based on GAN in its method

To verify the reasonableness of the optimal source domain selection, we used the citrus(orange) dataset as the source domain in the comparison algorithm. From Fig. 7, we can see that the distance of pear from apple as well as tomato is closer than the distance of citrus from apple and tomato. 

**Fig. 10.**
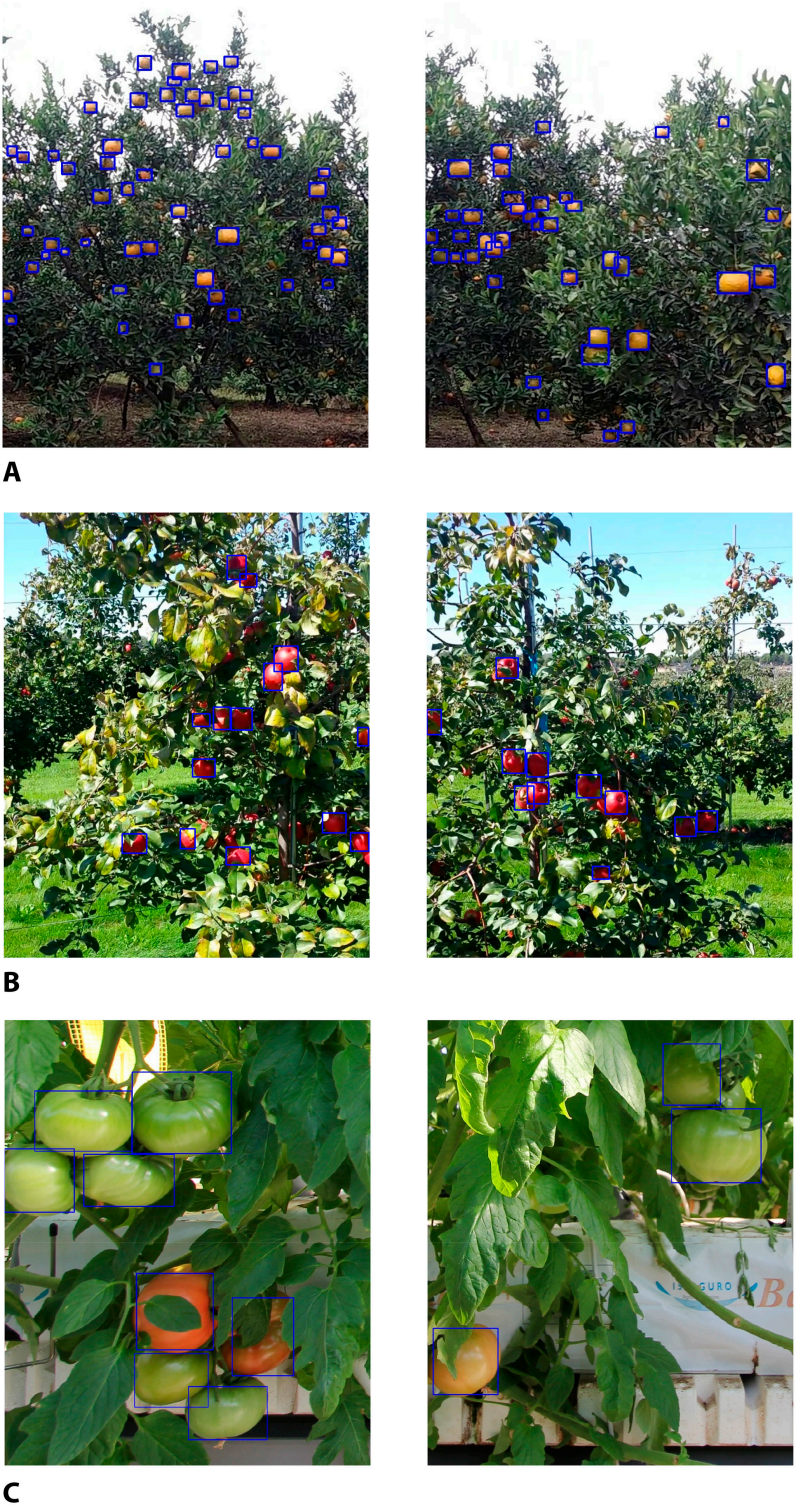
Visual images of the effect of citrus, apple, and tomato label generation. (A) Citrus orchard, (B) apple orchard, and (C) tomato orchard.

In this study, the images obtained from the synthetic dataset construction method using the target domain were pretrained for the CenterNet detection model. The prediction box was noise-filtered and cyclically updated using a pseudo-label self-learning method, which showed good performance in previous research [7]. The final target domain fruit autolabeling models *M_orange_*, *M_apple_*, *M_tomato_* were obtained to further improve the label generation accuracy while eliminating false detection frames. Figure 10 shows the label visualization results for the 3 fruit datasets.

Table 2 shows the results of a comparison between the proposed method and the current automatic labeling methods, respectively, traditional pseudo-label method (T-PL) [42], pseudo-label self-learning method (PL-SL) [43], and EasyDAM_V1 [6]. The following results were analyzed and discussed. The datasets used in the experiment are highly diverse, which citrus and apple images were collected from outdoor scenes while tomato images were collected from indoor scenes. The differences in shooting locations and shooting angles resulted in large variations in the scene and fruit size in the images. Moreover, most tomato images included green tomato fruit targets. Therefore, their color characteristics were similar to those of the background foliage. These differences in the acquisition environments, locations, and shooting distances presented challenges for this study. In the different experiments, the proposed method showed different degrees of improvement in precision rate, recall rate, and average precision compared with the previously reported methods. The average precision of the proposed method for the pear2apple and pear2tomato experiments was 89.78% and 90.84%, respectively, which represented increases of 0.08% and 8.54%, respectively, compared with EasyDam_V1, while the average precision of the pear2orange experiment reached 90.94%, which was the highest among the 3 datasets. Therefore, the proposed method is suitable for practical applications.

**Table 2.** Final label generation results obtained from the EasyDAM_V3 model compared with the EasyDAM_V1 model.

Experiment	Test set	Method	Precision↑	Recall↑	mAP↑
*Pear*2*orange*	Actual orchard orange images	Proposed	0.8846	0.8846	0.9094
*Pear*2*apple*	Actual orchard apple images	T-PL	0.803	0.808	0.852
		PL-SL	0.828	0.836	0.875
		EasyDAM_V1 method	0.836	0.834	0.897
		Proposed	0.8819	0.8819	0.8978
*Pear*2*tomato*	Actual orchard tomato images	T-PL	0.770	0.769	0.752
		PL-SL	0.769	0.767	0.769
		EasyDAM_V1 method	0.790	0.786	0.823
		Proposed	0.8685	0.8654	0.9084
Experiment	Test set	Method	Precision↑	Recall↑	mAP↑
*Pear*2*orange* − *exp* 2	Actual orchard orange images	Proposed	0.8846	0.8846	0.9094
*Pear*2*apple* − *exp* 2	Actual orchard apple images	T-PL	0.803	0.808	0.852
		PL-SL	0.828	0.836	0.875
		EasyDAM_V1 method	0.836	0.834	0.897
		Proposed	0.8819	0.8819	0.8978
*Pear*2*tomato* − *exp* 2	Actual orchard tomato images	T-PL	0.770	0.769	0.752
		PL-SL	0.769	0.767	0.769
		EasyDAM_V1 method	0.790	0.786	0.823
		Proposed	0.8685	0.8654	0.9084

In addition, a substantial improvement in the average accuracy in the *Pear*2*tomato*^2^ experiment was achieved using the proposed method in this paper, which was mainly because of the relatively higher resolution, fruit size, and pixel share of the fruit targets in the images from the tomato dataset. Moreover, the number of fruits in each image was small (as shown in Fig. 2C). In contrast, the opposite fruit distribution characteristics were obtained for the citrus and apple datasets. If the original EasyDAM_V1 method is used to extract the target domain fruit features directly using the source domain orchard images, then the different fruit distribution characteristics in different scenes will result in large domain differences, which will lead to a lower label generation quality. In this study, a synthetic dataset was used to artificially control the synthetic fruit size, number, and other information, and the anchor-free detector adaptively selected the most suitable anchor box scale. These characteristics resolved the domain differences and the degraded detection performance caused by different fruit distribution characteristics in different scenes. Meanwhile, to verify the effectiveness of each component method proposed in this paper as well as the actual performance, we also conducted ablation experiments. Details are given in Supplementary Materials. Thus, the proposed method significantly improves the accuracy and can be generalized to different types of fruit label generation tasks.

## Discussion

Based on previously presented research, we propose a new automatic labeling algorithm, EasyDAM_V3, for fruit datasets corresponding to multiple categories. The results show that the average precision of EasyDAM_V3 in automatic labeling of multiclass target domain fruit images is around 90% in different datasets. The difference in the average precision of automatic labeling between different datasets is only 1.16%, which can be seen that the proposed method performs more stable compared to the baseline method of 7.4%. In addition, compared with the previous study, the method in this paper has less impact on the model performance, more scene generalization, and better performance under different angles, shooting distances, and environments.

To validate the optimal source domain, this study selected different types of fruit datasets in the same cluster for experimental design and method validation. We selected tomatoes, apples, and oranges as the target domains and pear_2(white) as the optimal source domain using the optimal source domain selection method. Figure 7 shows that pitaya, cucumber, and banana have richer appearance features; thus, the distribution of these fruits in the multidimensional feature space was more discrete, even for the same datasets. Under such conditions, the most suitable fruit in the optimal source domain cannot be selected; as such, the method can only select the nearest fruits for fruit dataset translation and label generation. However, a single-source domain fruit cannot easily correspond to multiple species of target domain fruit images at the same time; therefore, for cases in which the most suitable source domain fruit cannot be selected, target domain fruit images with large phenotypic differences and realistic images should be generated by an image translation model, which will be the focus of our future research. In addition, we will improve the construction methods for synthetic datasets. A more realistic orchard scene in the target domain was obtained using depth information and 3D model construction. Methodological investigations and experimental designs for fruit growth subrules and branch and leaf colonization subrules will be carried out to further improve the intelligence and accuracy of the automatic labeling method. Furthermore, in the future, we will describe in more detail the different fruits within the same species, so that the traits of different fruits of the same variety can be analyzed and discussed.

In summary, the proposed automatic fruit labeling method based on optimal source domain selection can significantly improve the applicability and domain adaptability of automatic labeling algorithms. It can also achieve accurate and stable performance in datasets with large scene and fruit distribution characteristic differences. In addition, we hope to provide an effective method for selecting source domain datasets based on studies related to image translation and generate more realistic target domain images while achieving a better performance in downstream tasks.

## Data Availability

The data and executable files used in this paper will be available upon request at: https://github.com/I3-Laboratory/EasyDAM_V3_dataset.

## Supplementary Materials

Supplementary 1 Ablation experiment Figs. S1 to S5 Tables S1 to S4

## References

[1] Neupane C, Koirala A, Wang Z, Walsh KB. Evaluation of depth cameras for use in fruit localization and sizing: Finding a successor to Kinect v2. Agronomy. 2021;11(9):1780.

[2] Liu Y, Gao P, Zheng C, Tian L, Tian Y. A deep reinforcement learning strategy combining expert experience guidance for a fruit-picking manipulator. Electronics. 2022;11(3):311.

[3] He L, Fang W, Zhao G, Wu Z, Fu L, Li R, Majeed Y, Dhupia J. Fruit yield prediction and estimation in orchards: A state-of-the-art comprehensive review for both direct and indirect methods. Comput Electron Agric. 2022;195: 106812.

[4] Dewi T, Mulya Z, Risma P, Oktarina Y. BLOB analysis of an automatic vision guided system for a fruit picking and placing robot. Int J Comput Vis Robot. 2021;11(3):315–327.

[5] Koh JCO, Spangenberg G, Kant S. Automated machine learning for high-throughput image-based plant phenotyping. Remote Sens. 2021;13(5):858.

[6] Zhang W, Chen K, Wang J, Shi Y, Guo W. Easy domain adaptation method for filling the species gap in deep learning-based fruit detection. Hortic Res. 2021;8:119. 34059636 10.1038/s41438-021-00553-8 PMC8167097

[7] Zhang W, Chen K, Zheng C, Liu Y, Guo W. EasyDAM_V2: Efficient data labeling method for multishape, cross-species fruit detection. Plant Phenomics. 2022;2022:9761674. 36204392 10.34133/2022/9761674 PMC9513831

[8] Dash A, Ye J, Wang G. A review of Generative Adversarial Networks (GANs) and its applications in a wide variety of disciplines -- from Medical to Remote Sensing. arXiv. 2021. 10.48550/arXiv.2110.01442

[9] Isola P, Zhu JY, Zhou T, Efros AA. Image-to-image translation with conditional adversarial networks. Paper presented at IEEE: Proceedings of the 2017 IEEE Conference on Computer Vision and Pattern Recognition (CVPR); 2017 July 21–26; Honolulu, HI.

[10] Zhang W, Wang X, Tang X. Coupled information-theoretic encoding for face photo-sketch recognition. Paper presented at IEEE: Proceedings of the Computer Vision and Pattern Recognition (CVPR); 2011 June 20–25; Colorado Springs, CO.

[11] Eitz M, Hays J, Alexa M. How do humans sketch objects? ACM Trans Graph. 2012;31(4):1–10.

[12] Yi Z, Zhang H, Tan P, Gong M. DualGAN: Unsupervised dual learning for image-to-image translation. Paper presented at IEEE: Proceedings of the 2017 IEEE International Conference on Computer Vision (ICCV); 2017 October 22–29; Venice, Italy.

[13] Wang X, Tang X. Face photo-sketch synthesis and recognition. IEEE Trans Pattern Anal Mach Intell. 2009;31(11):1955–1967. 19762924 10.1109/TPAMI.2008.222

[14] Laffont P-Y, Ren Z, Tao X, Qian C, Hays J. Transient attributes for high-level understanding and editing of outdoor scenes. ACM Trans Graph. 2014;33(4):1–11.

[15] Zhu J-Y, Park T, Isola P, Efros AA. Unpaired image-to-image translation using cycle-consistent adversarial networks. Paper presented at IEEE: Proceedings of the 2017 IEEE International Conference on Computer Vision; 2017 October 22–29; Venice, Italy.

[16] Sun P, Kretzschmar H, Dotiwalla X, Chouard A, Patnaik V, Tsui P, Guo J, Zhou Y, Chai Y, Caine B, et al. Scalability in perception for autonomous driving: Waymo open dataset. Paper presented at IEEE: Proceedings of the 2020 IEEE/CVF Conference on Computer Vision and Pattern Recognition (CVPR); 2020.

[17] Zhu J-Y, Park T, Isola P, Efros AA. Unpaired image-to-image translation using cycle-consistent adversarial networks. Paper presented at IEEE: Proceedings of the 2017 IEEE International Conference on Computer Vision (ICCV); 2017 October 22–29; Venice, Italy.

[18] Huang X, Liu M, Belongie S, Kautz J. Multimodal unsupervised image-to-image translation. Proceedings of the European conference on computer vision (ECCV), Ferrari V, Hebert M, Sminchisescu C, Weiss Y, Eds. Springer, Cham; 2018; p. 179–196.

[19] Nie X, Ding H, Qi M, Wang Y, Wong EK. URCA-GAN: UpSample residual channel-wise attention generative adversarial network for image-to-image translation. Neurocomputing. 2021;443:75–84.

[20] Liu Z, Luo P, Wang X, Tang X. Deep learning face attributes in the wild. Paper presented at IEEE: Proceedings of the 2015 IEEE International Conference on Computer Vision (ICCV); 2015 December 7–13; Santiago, Chile.

[21] Langner O, Dotsch R, Bijlstra G, Wigboldus DHJ, Hawk ST, van Knippenberg A. Presentation and validation of the radboud face database. Cognit Emot. 2010;24(8):1377–1388.

[22] Choi Y, Uh Y, Yoo J, Ha J-W. StarGAN v2: Diverse image synthesis for multiple domains. Paper presented at IEEE: Proceedings of the 2020 IEEE/CVF Conference on Computer Vision and Pattern Recognition (CVPR); 2020 June 13–19; Seattle, WA.

[23] Liu J, Qu F, Hong X, Zhang H. A small-sample wind turbine fault detection method with synthetic fault data using generative adversarial nets. IEEE Trans Industr Inform. 2018;15(7):3877–3888.

[24] Gao H, Zhang Y, Lv W, Yin J, Qasim T, Wang D. A deep convolutional generative adversarial networks-based method for defect detection in small sample industrial parts images. Appl Sci. 2022;12(13):6569.

[25] Li C, Zhang Y, Qu Y. Object detection based on deep learning of small samples. Paper presented at IEEE: Proceedings of the 2018 Tenth International Conference on Advanced Computational Intelligence (ICACI); 2018 March 29–31; Xiamen, China.

[26] Janoch A, Karayev S, Jia Y, Barron JT, Fritz M, Saenko K, Darrell T. A category-level 3D object dataset: Putting the Kinect to work. Paper presented at: 2011 IEEE International Conference on Computer Vision Workshops (ICCV Workshops);2011 November 6–13; Barcelona, Spain.

[27] Hu X-D, Xq W, Meng F-J, Hua X, Yan Y-J, Li Y-Y, Huang J, Xl J. Gabor-CNN for object detection based on small samples. Def Technol. 2020;16(6):1116–1129.

[28] Abbas A, Jain S, Gour M, Vankudothu S. Tomato plant disease detection using transfer learning with C-GAN synthetic images. Comput Electron Agric. 2021;187: 106279.

[29] Skovsen S, Dyrmann M, Mortensen AK, Steen KA, Green O, Eriksen J, Gislum R, Jørgensen RN, Karstoft H. Estimation of the botanical composition of clover-grass leys from RGB images using data simulation and fully convolutional neural networks. Sensors. 2017;17(12):2930. 29258215 10.3390/s17122930 PMC5751073

[30] Skovsen S, Dyrmann M, Eriksen J, Gislum R, Karstoft H, Jørgensen RN. Predicting dry matter composition of grass clover leys using data simulation and camera-based segmentation of field canopies into white clover, red clover, grass and weeds. Paper presented at: Proceedings of the 14th International Conference on Precision Agriculture; International Society of Precision Agriculture; 2018 June 24–27; Montréal, Canada; vol. 2.

[31] Skovsen S, Dyrmann M, Mortensen AK, Laursen MS, Gislum R, Eriksen J, Farkhani S, Karstoft H, Jørgensen RN. The GrassClover image dataset for semantic and hierarchical species understanding in agriculture. Paper presented at IEEE: Proceedings of the 2019 IEEE/CVF Conference on Computer Vision and Pattern Recognition Workshops (CVPRW); June 16–17; Long Beach, CA.

[32] Ros G, Sellart L, Materzynska J, Vazquez D, Lopez AM. The SYNTHIA dataset: A large collection of synthetic images for semantic segmentation of urban scenes. Paper presented at IEEE: Proceedings of the 2016 IEEE Conference on Computer Vision and Pattern Recognition (CVPR); 2016 June 27–30; Las Vegas, NV.

[33] Thompson A. Fruits 360 dataset. 2017. [accessed 28 August 2018] https://www.kaggle.com/moltean/fruits vol. 02.

[34] Hani N, Roy P, Isler V. Minneapple: A benchmark dataset for apple detection and segmentation. IEEE Robot Autom Lett. 2020;5(2):852–858.

[35] Mu Y, Chen TS, Ninomiya S, Guo W. Intact detection of highly occluded immature tomatoes on plants using deep learning techniques. Sensors. 2020;20(10):2984. 32466108 10.3390/s20102984 PMC7288109

[36] Yamamoto K, Ninomiya S, Kimura Y, Hashimoto A, Yoshioka Y, Kameoka T. Strawberry cultivar identification and quality evaluation on the basis of multiple fruit appearance features. Comput Electron Agric. 2015;110:233–240.

[37] Li M, Coneva V, Robbins KR, Clark D, Chitwood D, Frank M. Quantitative dissection of color patterning in the foliar ornamental coleus. Plant Physiol. 2021;187(3):1310–1324. 34618067 10.1093/plphys/kiab393 PMC8566300

[38] Chen J, Kellokumpu V, Zhao G, Pietikäinen M. RLBP: Robust local binary pattern. Paper presented at: Proceedings of the British Machine Vision Conference (BMVC 2013); Bristol, UK; 2013.

[39] Zhou X, Wang D, Krähenbühl P. Objects as points. arXiv. 2019. 10.48550/arXiv.1904.07850

[40] Redmon J, Farhadi A. YOLOv3: An incremental improvement. arXiv. 2018. 10.48550/arXiv.1804.02767

[41] Bochkovskiy A, Wang C-Y, Liao H-YM. YOLOv4: Optimal speed and accuracy of object detection. arXiv. 2020. 10.48550/arXiv.2004.10934

[42] Liu Y-C, Ma C-Y, He Z, Kuo C-W, Chen K, Zhang P, Wu B, Kira Z, Vajda P. Unbiased teacher for semi-supervised object detection. arXiv. 2021. 10.48550/arXiv.2102.09480

[43] Sohn K, Zhang Z, Li C-L, Zhang H, Lee C-Y, Pfister T. A simple semi-supervised learning framework for object detection. arXiv. 2020. 10.48550/arXiv.2005.04757

