# Supplementary material for: EasyDAM_V3: Automatic Fruit Labeling Based on Optimal Source Domain Selection and Data Synthesis via a Knowledge Graph
Source: Plant Phenomics. 2023 Jul 27;5:0067. doi: 10.34133/plantphenomics.0067 (PMC10374194; doi:10.34133/plantphenomics.0067)
Supplement: Supplementary 1 — Ablation experiment Figs. S1 to S5 Tables S1 to S4 [file plantphenomics.0067.f1.docx]

Supplementary Materials

## **Ablation experiment**

**Validation of the target domain synthetic dataset construction module:**

Table 3 shows the training of the CenterNet detection model for images obtained using only the synthetic dataset construction method in the target domain. The detection results were tested in actual target domain images, and we then performed a comparative analysis. The detection models for all three types of fruit trained using only synthetic datasets achieved more than 70% accuracy in actual scenes in the target domain. This provided a high-quality pretrained model for subsequent pseudo-label loop updates. The average precision of the ${Pear2apple}^{1}$ experiment using the synthetic dataset was 71.73%, which represented a 6.43% improvement compared with the EasyDAM_V1 method. The average precision of the ${Pear2tomato}^{1}$ experiment was 72.93%, which represented a 1.83% improvement compared with the EasyDAM_V1 method. Moreover, the precision and recall rates were both improved to different degrees as well.

In addition, because the EasyDAM_V1 method uses citrus as the source domain, a cross-sectional comparison of the detection accuracy of the citrus dataset cannot be performed. However, we found that the average precision of the ${Pear2orange}^{pre}$ experiment for citrus in the synthetic dataset was 77.15%. The longitudinal comparisons of the tomato and apple experiments can also indirectly verify the effectiveness of the synthetic dataset method proposed in this study. Meanwhile, the spatial distribution of multi-dimensional features of fruits in Figure 7 shows that the distance between the phenotypic features of apple and citrus is smaller than that between apple and pear. Theoretically, the accuracy of apple image detection should be higher when orange is used as the source domain. However, this study used pear_2(white) as the source domain and a synthetic dataset as the training set, and the detection performance of the proposed method in the ${Pear2apple}^{pre}$ experiment was better than that of the EasyDAM_V1 method. Thus, the effectiveness of synthetic dataset construction methods for training high-performance fruit detection models was revealed.

**Table S1.** Performance of the training models obtained using two different methods in different orchards.

| **Experient** | **Test set** | **Training set** | **Precision↑** | **Recall↑** | **mAP↑** |
| --- | --- | --- | --- | --- | --- |
| ${Pear2orange}^{pre}$ | Actual orchard orange images | **Synthetic dataset** | **0.7427** | **0.7438** | **0.7715** |
| ${Pear2apple}^{pre}$ | Actual orchard apple images | EasyDAM dataset | 0.704 | 0.658 | 0.653 |
|  |  | **Synthetic dataset** | **0.7093** | **0.6969** | **0.7173** |
| ${Pear2tomato}^{pre}$ | Actual orchard tomato images | EasyDAM dataset | 0.723 | 0.725 | 0.711 |
|  |  | **Synthetic dataset** | **0.7369** | **0.7369** | **0.7293** |

Note: Because the EasyDAM method selects citrus as the source domain dataset, its performance in citrus images could not be obtained.

**Component ablation experiment:**

To verify the effectiveness of each component of the method in the synthetic dataset building module of this study and compare the performance of the different component methods, component ablation experiments were performed. In the initial case, this study used the method of randomly placing simulated target domain fruits in the background of the target domain for the construction of synthetic datasets. Based on this, we added illumination and size subrule $S_{2}$, graded obscuration subrule $G_{3}$, and natural semantic-based composition rule $N$. A synthetic dataset was constructed to gradually approximate the real orchard scene. Label generation was tested separately. In this study, the citrus, tomato, and apple datasets were evaluated using three metrics, namely precision, recall, and average precision. The results are presented in Tables 4–6.

The results for the citrus, tomato, and apple datasets showed that the average accuracy of label generation was improved by 4.58%, 7.74%, and 2.43% by adding leaf and fruit illumination and size subrule $S_{2}$, improved by 4.76%, 1.2%, and 8.38% by adding graded obscuration subrule $G_{3}$, and improved by 0.28%, 1.83%, and 2.05% by adding the natural semantic-based composition rule $N$, respectively. The results also showed that the average precision of label generation was improved to different degrees with the addition of synthetic dataset composition rules. Adding the illumination and size subrule $S_{2}$ for leaves and fruits significantly improved the average precision in the citrus and tomato datasets, although only a limited improvement was observed for the apple dataset. This is because the light intensity applied to the targets in the apple dataset was relatively strong. Light environments are not very diverse, and they are mostly in bright light environments (as shown in Figure 2(b)). Therefore, the performance improvement of this method in the apple dataset was not significant. Relevant methodological investigations and experimental comparisons of fruit growth subrules and branch colonization subrules will be performed in our subsequent work.

**Table S2.** Component ablation experiments using the citrus dataset

| **Method** | Illumination and size subrule $S_{2}$ | Graded obscuration subrule $G_{3}$ | Natural semantic-based composition rule $N$ | **P** | **R** | **AP** |
| --- | --- | --- | --- | --- | --- | --- |
| Construction of synthetic datasets |  |  |  | 0.7963 | 0.7963 | 0.8132 |
|  | **√** |  |  | 0.8428 | 0.8428 | 0.8590 |
|  | **√** | **√** |  | 0.9036 | 0.9036 | 0.9066 |
|  | **√** | **√** | **√** | 0.8846 | 0.8846 | 0.9094 |

**Table S3.** Component ablation experiments using the tomato dataset

| **Method** | Illumination and size subrule $S_{2}$ | Graded obscuration subrule $G_{3}$ | Natural semantic-based composition rule $N$ | **P** | **R** | **AP** |
| --- | --- | --- | --- | --- | --- | --- |
| Construction of synthetic datasets |  |  |  | 0.7781 | 0.7782 | 0.8007 |
|  | **√** |  |  | 0.8709 | 0.8680 | 0.8781 |
|  | **√** | **√** |  | 0.8534 | 0.8382 | 0.8901 |
|  | **√** | **√** | **√** | 0.8685 | 0.8654 | 0.9084 |

**Table S4.** Component ablation experiments using the apple dataset

| **Method** | Illumination and size subrule $S_{2}$ | | Graded obscuration subrule $G_{3}$ | Natural semantic-based composition rule $N$ | **P** | **R** | **AP** |  |
| --- | --- | --- | --- | --- | --- | --- | --- | --- |
| Construction of synthetic datasets |  |  | |  | 0.7340 | 0.7340 | 0.7692 | |
|  | **√** |  | |  | 0.7592 | 0.7582 | 0.7935 | |
|  | **√** | **√** | |  | 0.8735 | 0.8735 | 0.8773 | |
|  | **√** | **√** | | **√** | 0.8819 | 0.8819 | 0.8978 | |


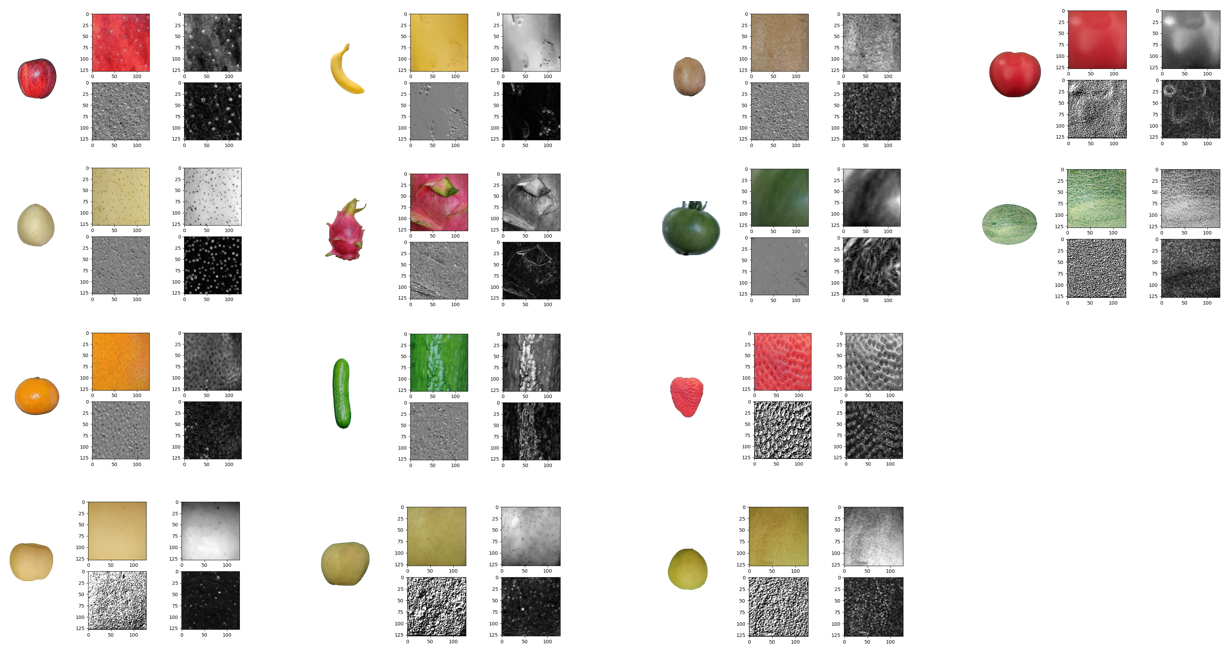


**Fig. S1** Visualization feature results of LBP descriptors.


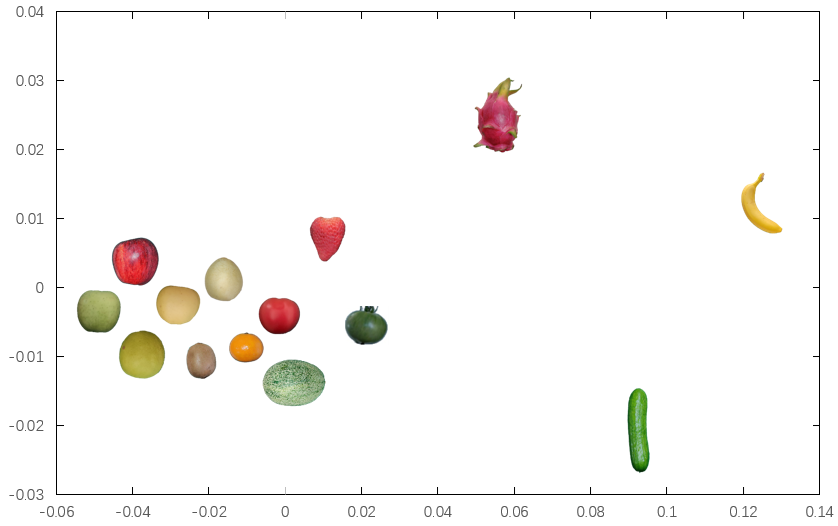


**Fig. S2** Spatial reconstruction of fruit shape features obtained by Fourier descriptors.


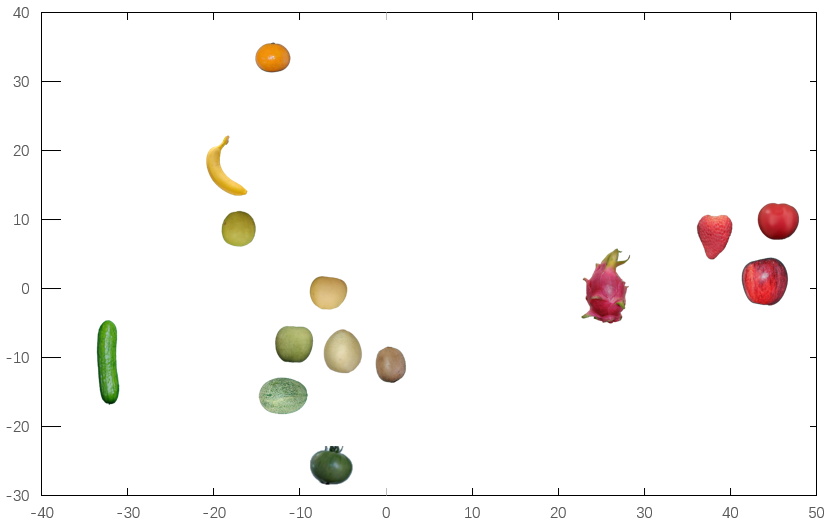


**Fig. S3** Spatial reconstruction of fruit color features obtained by color histogram.


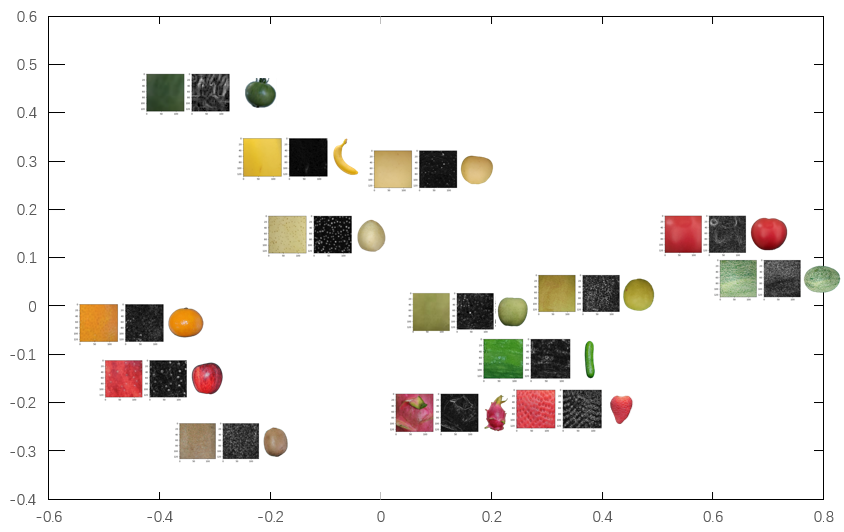


**Fig. S4** Spatial reconstruction of fruit texture features obtained by LBP descriptors.


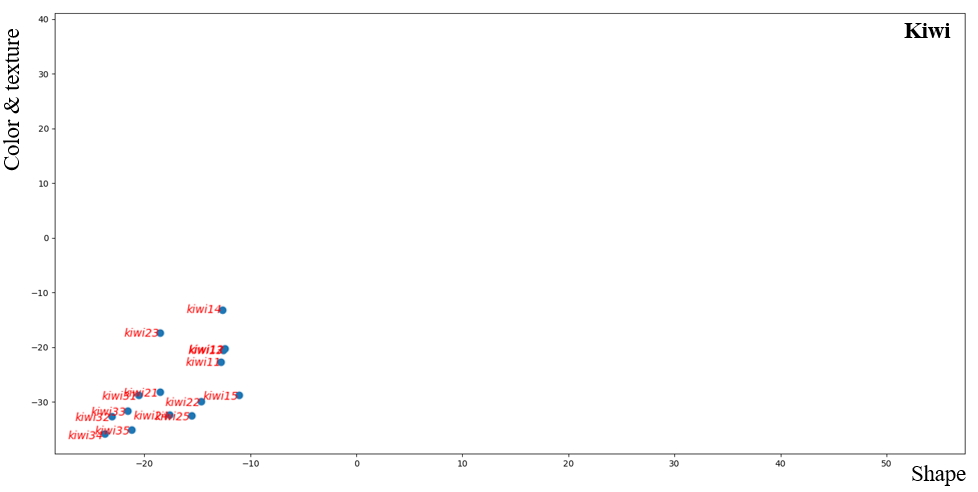


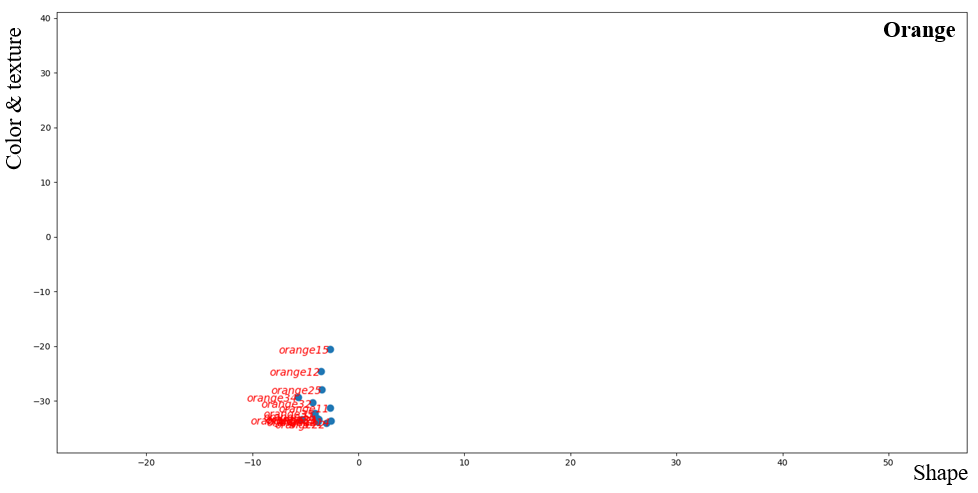


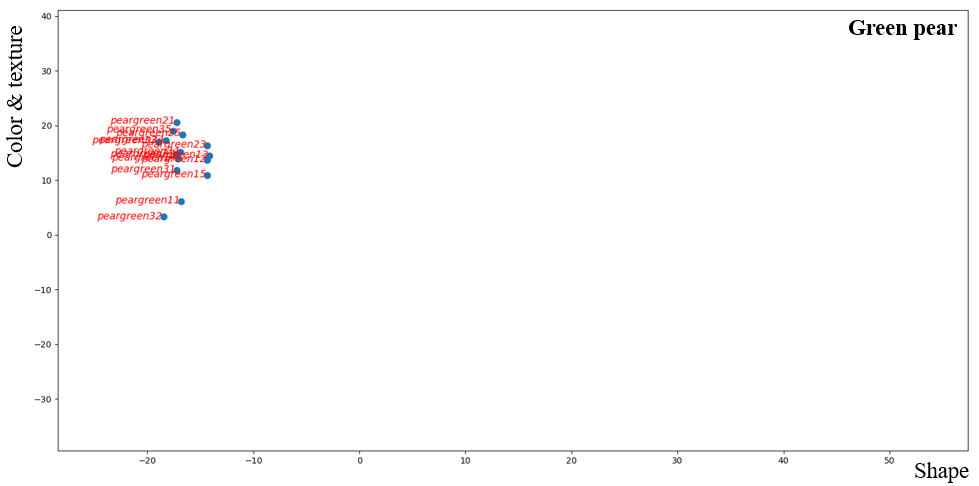


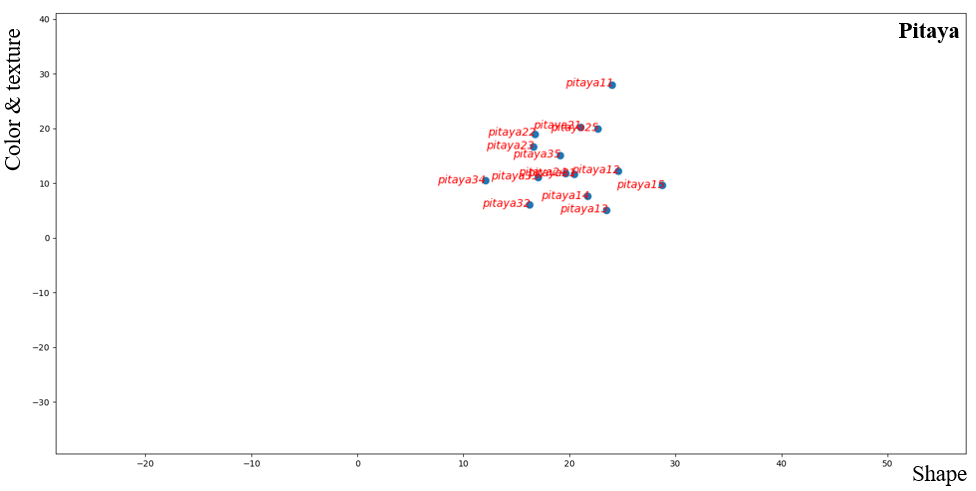


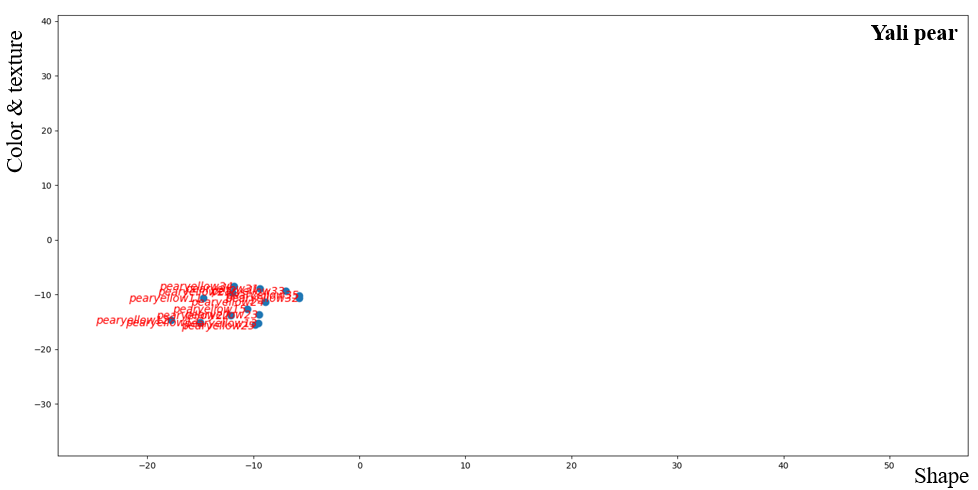


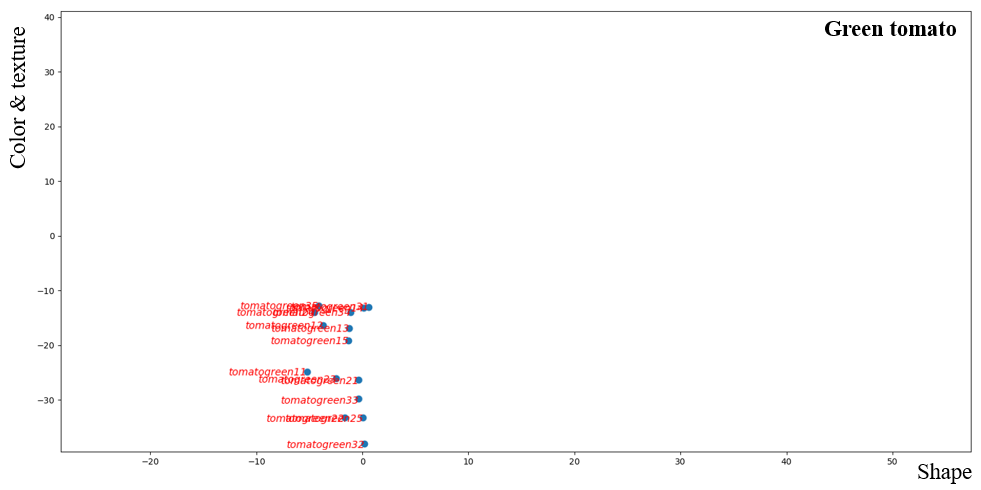


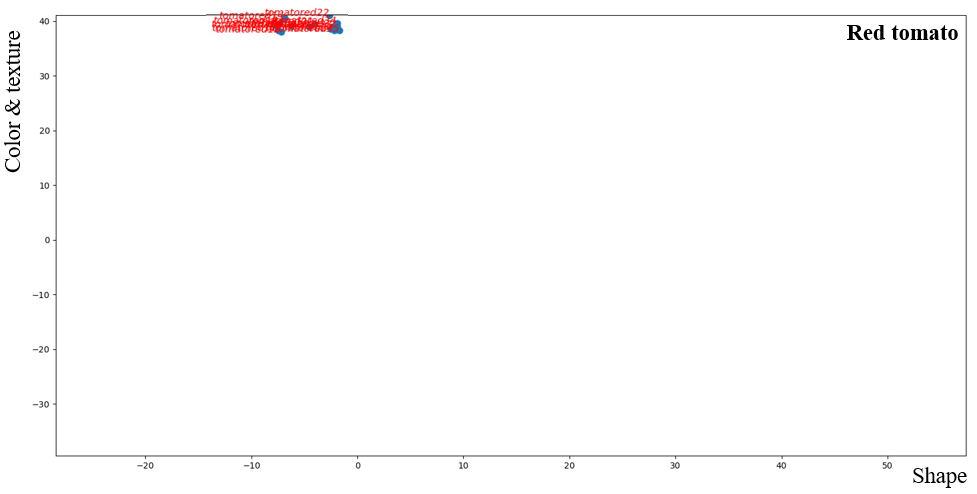


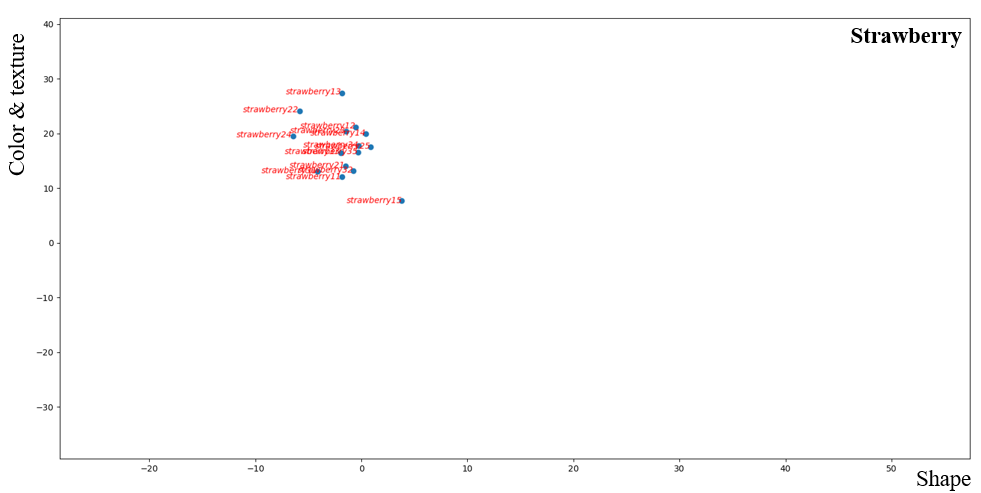


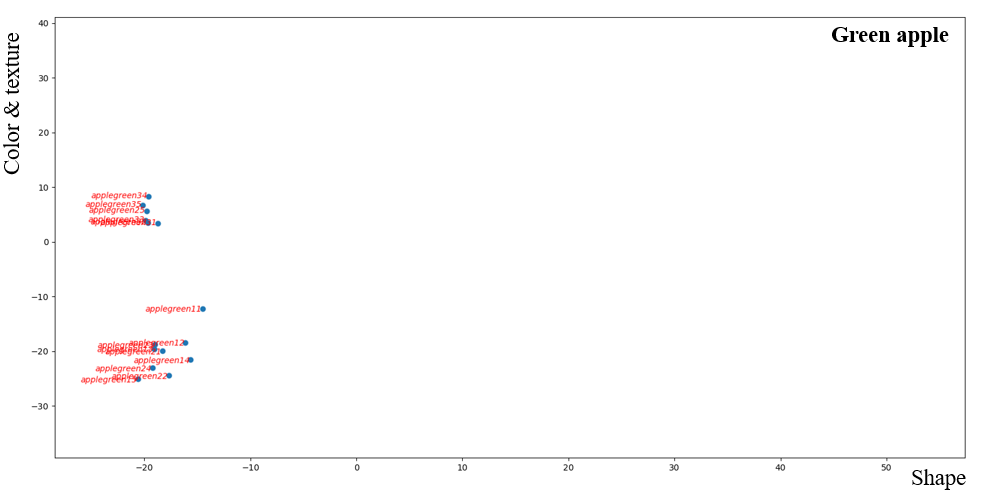


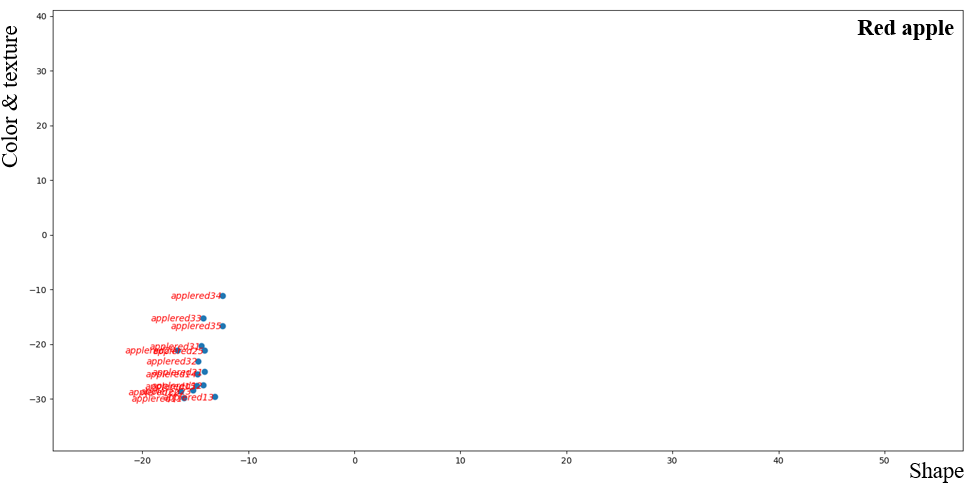


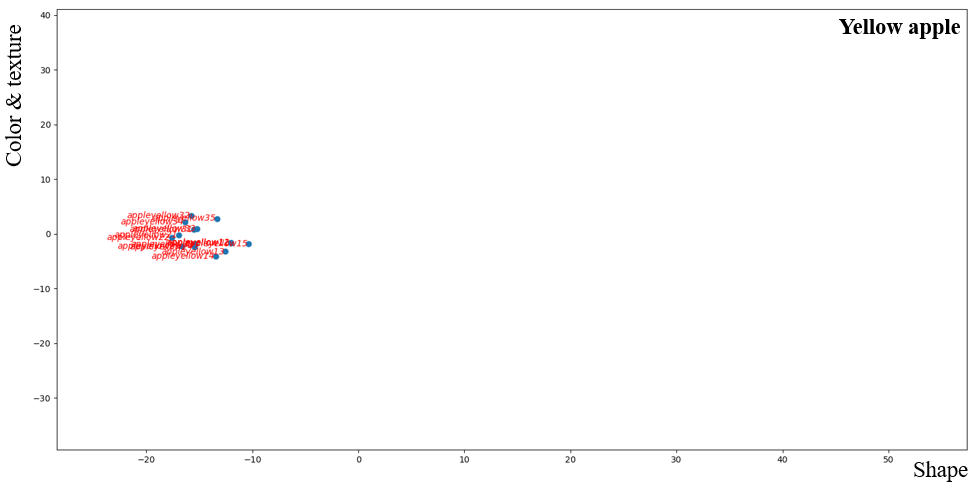


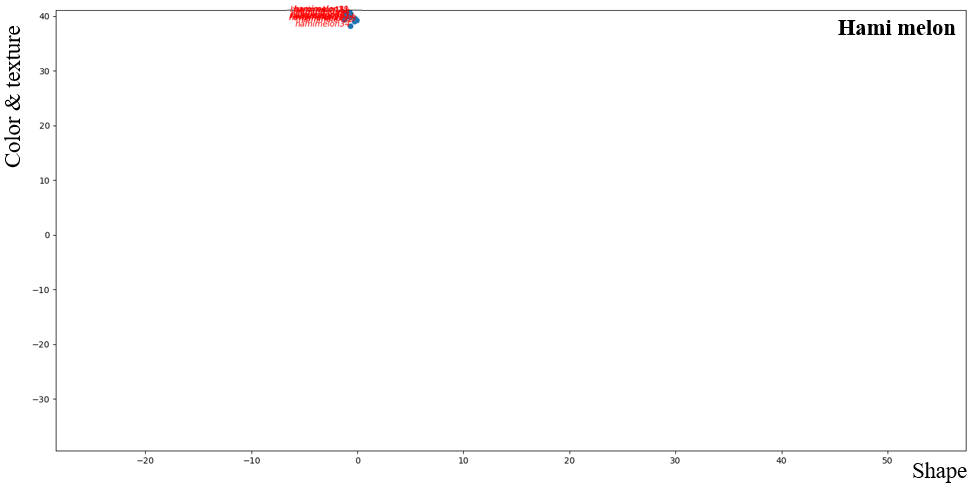


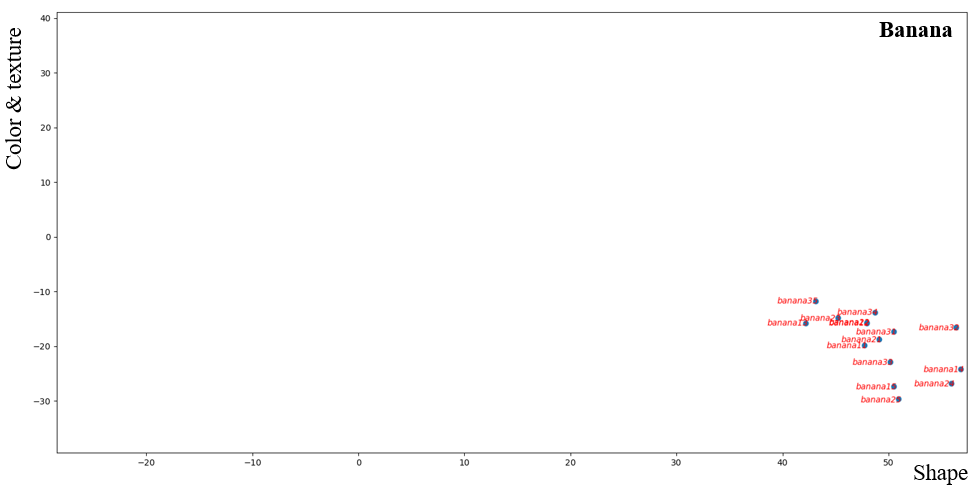


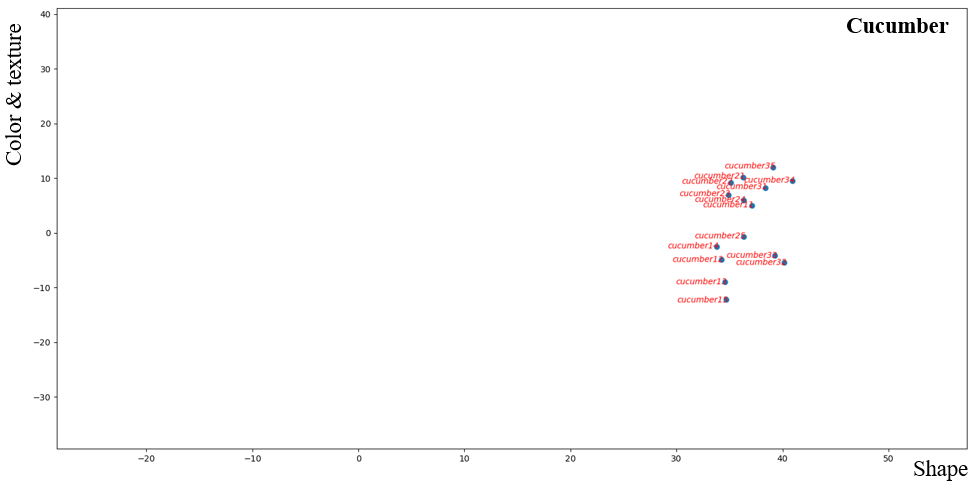


**Fig. S5** Position of different samples of each dataset of fruit in the multi-dimensional feature space reconstruction map.
